# CIRP Promotes Redox-Inflammatory Endothelial Injury in High-Fat Diet-Fed ApoE^−/−^ Mice and HUVECs: Involvement of TLR4/SIRT6 Signaling

**DOI:** 10.3390/antiox15080943

**Published:** 2026-07-29

**Authors:** Danli Chen, Jianjun Yang, Lingxuan Ren, Zihan Zheng, Zhen Jin, Jianli Gu, Nanbo Zheng, Weirong Wang, Jianyu He, Rong Lin

**Affiliations:** 1 Department of Pharmacology, School of Basic Medical Sciences, Xi’an Jiaotong University Health Science Center, Xi’an 710061, China; 2 Institute of Cardiovascular Science, Xi’an Jiaotong University Health Science Center, Xi’an 710061, China; 3 The First Affiliated Hospital, Yangtze University, Jingzhou 434000, China; 4 Laboratory Animal Center, Xi’an Jiaotong University, Xi’an 710061, China

**Keywords:** CIRP, SIRT6, TLR4, oxidative stress, endothelial dysfunction, eNOS acetylation, atherosclerosis, redox imbalance

## Abstract

Cold-inducible RNA-binding protein (CIRP/CIRBP) is recognized as an extracellular damage-associated molecular pattern. However, its relationship to endothelial redox-inflammatory injury in atherosclerosis remains poorly characterized. Here, we explored the associations among CIRP, endothelial dysfunction, and TLR4-/SIRT6-related changes. In high-fat diet (HFD)-fed ApoE^−/−^ mice, circulating CIRP was elevated and positively correlated with atherosclerotic plaque burden. This increase coincided with systemic redox imbalance, impaired NO/eNOS activity, and vascular inflammation. In HUVECs, CIRP exposure reduced cell viability and impaired NO/eNOS function. These effects were accompanied by increased ROS accumulation and MDA content, together with reduced GSH-Px activity. CIRP also increased inflammatory cytokine production, NF-κB p65 phosphorylation, and THP-1 adhesion. At the molecular level, CIRP reduced SIRT6 expression. Overexpression of SIRT6 attenuated CIRP-induced endothelial injury, oxidative stress, and NO/eNOS dysfunction. CIRP also increased TLR4 expression. Accordingly, pharmacological TLR4 inhibition with TAK-242 attenuated CIRP-associated endothelial injury and partially restored SIRT6 expression and protein stability. Conversely, SIRT6 silencing weakened the protective effects of TAK-242. In addition, CIRP exposure was accompanied by increased overall eNOS acetylation. This increase was attenuated by TAK-242 and further enhanced by SIRT6 silencing. Exploratory cross-context transcriptomic analysis identified overlapping inflammatory and oxidative stress-related signatures. Representative antioxidant-related changes were further examined in CIRP-treated HUVECs through assessment of GPX4 and CAT mRNA expression and CAT activity. Finally, in a preliminary clinical cohort, serum CIRP levels were higher in patients with coronary heart disease and were positively associated with Gensini score, including selected exploratory multivariable models. Overall, these findings provide preliminary evidence that CIRP is associated with redox-inflammatory endothelial injury. The results are also consistent with the possible involvement of TLR4-/SIRT6-related signaling. These observations should be interpreted cautiously because of the relatively high CIRP concentration used in vitro, the cross-context transcriptomic comparison, and the small clinical cohort.

## 1. Introduction

Cold-inducible RNA-binding protein (CIRBP/CIRP) is a glycine-rich RNA-binding protein initially identified as an intracellular stress-responsive molecule [1]. Under inflammatory or ischemic stress, CIRP can be released into the extracellular space, where it functions as a damage-associated molecular pattern (DAMP) and promotes sterile inflammatory responses through pattern-recognition receptor-related signaling [2,3]. Against this background, emerging clinical and experimental evidence has linked extracellular CIRP to cardiovascular and vascular injury. In patients with acute coronary syndrome, higher plasma CIRP levels were associated with major adverse cardiovascular events during follow-up [4]. Similarly, elevated CIRP levels have also been associated with disease severity and prognosis after cardiac arrest [5]. Together, these clinical observations support further investigation of the potential role of CIRP in vascular injury. Experimental vascular studies provide additional evidence for this relationship. In an abdominal aortic aneurysm model, CIRP levels increased in circulation, whereas anti-CIRP treatment attenuated aneurysmal dilation, inflammatory mediator expression, and macrophage accumulation [6]. More recently, extracellular CIRP was reported to promote TLR4-related vascular inflammation and MMP-2 expression in aortic endothelial cells and to aggravate aortic dissection in susceptible mice [7]. At the cellular level, direct endothelial studies further support a relationship between CIRP and vascular dysfunction. Recombinant CIRP increased vascular leakage and leukocyte infiltration in vivo. In primary vascular endothelial cells, CIRP activated NADPH oxidase-related responses, increased oxidative stress, and promoted NLRP3 inflammasome activation [8]. In addition, extracellular CIRP enhanced neutrophil–endothelial interactions and trans-endothelial migration [9]. A more recent study using HUVEC monolayers showed that CIRP increased endothelial permeability and disrupted intercellular junctions, as reflected by altered VE-cadherin and β-catenin organization [10]. Together, these findings associate extracellular CIRP with vascular inflammation, oxidative stress, leukocyte recruitment, and endothelial dysfunction. However, these studies were largely conducted in acute injury models. Whether CIRP is associated with redox imbalance and endothelial dysfunction under atherosclerosis-related conditions remains unclear.

Redox imbalance and impaired NO/eNOS function are central features of endothelial injury. We therefore considered intracellular regulators of endothelial stress responses. SIRT6 is an NAD^+^-dependent deacetylase involved in inflammation, oxidative stress, and vascular homeostasis [11,12]. Experimental studies support a protective role for SIRT6 in the vascular endothelium. SIRT6-haploinsufficient mice, endothelium-dependent vasorelaxation was impaired, whereas inhibition of NADPH oxidase improved vascular function, suggesting an association between SIRT6 deficiency and redox-sensitive endothelial dysfunction [13]. In HUVECs, H_2_O_2_ reduced SIRT6 and eNOS expression, whereas SIRT6 overexpression attenuated endothelial senescence and dysfunction and eNOS activity [14]. Our previous work further linked SIRT6 to atherosclerosis-related endothelial stress. Endothelium-specific SIRT6 depletion in ApoE^−/−^ mice reduced aortic NO release and eNOS activity [15]. In HUVECs, cholesterol crystals suppressed SIRT6 expression and impaired NO/eNOS function, whereas SIRT6 overexpression attenuated these changes [16]. Similarly, in an ox-LDL-treated HUVEC model, reduced SIRT6 expression was accompanied by increased ROS and MDA levels and decreased SOD activity [17]. SIRT6 has also been reported to attenuate ox-LDL-induced endothelial pyroptosis and inflammatory injury [18]. Together, these findings position SIRT6 as a regulator of endothelial responses to atherosclerosis-related oxidative and sterile inflammatory stress. However, these observations were derived primarily from cholesterol crystal- and ox-LDL-based models. Whether extracellular CIRP similarly alters SIRT6 expression, and whether restoring SIRT6 attenuates CIRP-associated redox imbalance and endothelial dysfunction, remains unclear.

TLR4 is a transmembrane pattern-recognition receptor and one of the best-characterized receptors involved in extracellular CIRP signaling. Previous studies have shown that extracellular CIRP can interact with TLR4 and activate downstream inflammatory signaling [2,3]. More directly relevant to endothelial cells, TLR4 deficiency prevented CIRP internalization in pulmonary vascular endothelial cells, whereas TLR2 deficiency did not affect this process [19]. In a recent vascular disease study, extracellular CIRP increased iNOS and MMP-2 expression and promoted NLRP3 inflammasome activation in human aortic endothelial cells. Targeting extracellular CIRP also attenuated the exacerbation of aortic dissection in susceptible mice [7]. Together, these observations support the involvement of TLR4 in endothelial responses to extracellular CIRP. However, whether TLR4-associated signaling is linked to CIRP-related SIRT6 downregulation in endothelial cells remains unclear. Beyond its expression, the functional consequences of SIRT6 on eNOS have also been investigated. A recent study showed that SIRT6 interacts with eNOS and deacetylates Lys494, Lys497, and Lys504 within its calmodulin-binding domain. These changes promoted calmodulin binding, eNOS activity, and NO production [20]. This evidence suggests that SIRT6 may regulate endothelial function through both eNOS expression and post-translational modification. Nevertheless, it remains unknown whether CIRP exposure is accompanied by altered overall eNOS acetylation and whether this change is associated with TLR4-/SIRT6-related signaling.

Extracellular CIRP has been linked to TLR4-associated endothelial inflammation, whereas SIRT6 contributes to endothelial redox homeostasis and NO/eNOS regulation. However, the relationships among CIRP exposure, TLR4 signaling, SIRT6 expression, overall eNOS acetylation, and endothelial dysfunction remain unclear. Using HFD-fed ApoE^−/−^ mice, CIRP-stimulated HUVECs, exploratory transcriptomic analysis, and a preliminary cohort of patients with coronary heart disease, we investigated the association of CIRP with redox-inflammatory endothelial injury and explored the potential involvement of TLR4-/SIRT6-related signaling under atherosclerosis-related conditions.

## 2. Materials and Methods

### 2.1. Animals and Experimental Design

Male ApoE^−/−^ mice (8 weeks old, weighing 20–22 g) on a C57BL/6J background were purchased from the Laboratory Animal Management Committee of Xi’an Jiaotong University (Xi’an, China). All animals were housed in a specific pathogen-free (SPF) facility under controlled conditions (temperature 22 ± 2 °C, humidity 50–60%, 12 h light/dark cycle) with free access to standard chow and water. After a 1-week acclimatization period, mice were randomly assigned to the following experimental groups (*n* = 10 per group): (1) a baseline group (0 w); (2) an HFD group (12 w), which was fed an HFD containing 0.15% cholesterol and 21% fat (Keao Xieli, Beijing, China) for 12 weeks. The sample size was selected based on previous studies using similar ApoE^−/−^ mouse models and our preliminary experiments. At the end of the experimental period, mice were fasted for 12 h and were euthanized via overdose i.p. injections of pentobarbital. Blood samples were collected via cardiac puncture, allowed to clot at room temperature for 30 min, and centrifuged at 3000× *g* for 15 min at 4 °C. Serum was aliquoted and stored at −80 °C until analysis. The aorta was carefully dissected and rinsed with ice-cold PBS. The heart containing the aortic root was embedded in OCT compound and sectioned for H&E and Oil Red O staining. The remaining aortic tissues were collected for Western blotting, RT-qPCR, and biochemical assays. Images were assessed using a fully automated digital slide scanner. The area of atherosclerotic lesions was analyzed by ImageJ software (version 1.54). All animal procedures were approved by the Experimental Animal Ethics Committee of Xi’an Jiaotong University (approval number: 2023-1411) and conducted in accordance with the Guide for the Care and Use of Laboratory Animals (NIH Publication No. 85-23, revised 1996).

### 2.2. Clinical Sample Collection

This retrospective case–control clinical analysis included 21 patients with coronary heart disease (CHD) and 20 healthy examination controls. Because this was an observational study, random allocation was not applicable. Clinical sample size was estimated using the two-sided two-sample means calculator at PowerAndSampleSize.com (https://powerandsamplesize.com/, accessed on 28 March 2023) based on pilot data from 11 patients with CHD and 5 healthy controls (mean CIRP levels, 308.03 vs. 95.53; pooled SD, 39.82; α = 0.05; power = 0.90; allocation ratio, 1:1), yielding a minimum of three participants per group for the primary case–control comparison. Because exploratory analyses according to CHD severity were planned, 21 patients with CHD and 20 healthy controls were ultimately included; the subgroup analyses were considered exploratory. Patients with CHD were recruited from the Department of Cardiology, the First Affiliated Hospital of Xi’an Jiaotong University, between 28 March 2023 and 28 August 2023. CHD was diagnosed according to coronary angiography, with significant coronary artery disease defined as ≥50% luminal stenosis in at least one major coronary artery. Healthy controls were selected from individuals undergoing routine health examinations during the same period. Based on available medical records, physical examination, chest X-ray, and electrocardiography, these individuals had no known history of cardiovascular disease.

Coronary angiographic findings were assessed by two experienced interventional cardiologists. Coronary atherosclerotic burden was quantified using the Gensini score, with the detailed scoring criteria provided in Supplementary Table S1. Patients with CHD were stratified into low-score (0–27), intermediate-score (28–50), and high-score (≥51) groups according to their Gensini scores. Patients were eligible for inclusion if they were older than 18 years, met the clinical diagnostic criteria for CHD, had coronary artery lesions confirmed by angiography, and had complete clinical data. Exclusion criteria included hypertension, diabetes mellitus, severe respiratory disease, hematological disease, severe hepatic or renal dysfunction, autoimmune disease, malignant tumor, cardiomyopathy, aortic dissection, heart failure, persistent arrhythmia, cerebrovascular disease, pulmonary embolism, thyroid dysfunction, acute or chronic infection, mental or legal disability, incomplete clinical data, use of antibiotics, glucocorticoids, anti-inflammatory analgesics, or immunosuppressive agents within 4 weeks, and use of thrombolytic, anticoagulant, antiplatelet, or lipid-lowering drugs within 4 weeks.

Baseline clinical information was obtained from medical records, including age, sex, heart rate, blood pressure, serum creatinine, lipid profiles, and blood cell counts. Serum total cholesterol (TC), low-density lipoprotein cholesterol (LDL-C), and high-density lipoprotein cholesterol (HDL-C) were measured using an automated biochemical analyzer in the clinical laboratory of the First Affiliated Hospital of Xi’an Jiaotong University. Lymphocyte, monocyte, and neutrophil counts were measured using an automated hematology analyzer. The atherosclerosis index (AI), monocyte-to-HDL-C ratio (MHR), lymphocyte-to-HDL-C ratio (LHR), and neutrophil-to-HDL-C ratio (NHR) were calculated as follows: AI = (TC − HDL-C)/HDL-C; MHR = monocyte count/HDL-C; LHR = lymphocyte count/HDL-C; and NHR = neutrophil count/HDL-C. Peripheral venous blood samples were collected and allowed to stand at room temperature for 1 h. Serum was separated by centrifugation at 3000× *g* for 15 min and stored at −80 °C until analysis. Serum CIRP levels were measured using a commercial Human CIRBP ELISA kit (CSB-EL005440HU, CUSABIO, Wuhan, China) according to the manufacturer’s instructions. CIRP concentrations were calculated from the standard curve. All clinical data were anonymized before analysis. The study was conducted in accordance with the Declaration of Helsinki and was approved by the Medical Ethics Committee of the First Affiliated Hospital of Xi’an Jiaotong University (approval number: 2023-332). Written informed consent was obtained from all participants. Clinical trial registration was not applicable because this was a non-interventional observational study.

### 2.3. Cell Culture and Treatment

Human umbilical vein endothelial cells (HUVECs) were purchased from the American Type Culture Collection (CRL-1730, ATCC, Manassas, VA, USA) and cultured in endothelial cell medium (ECM, 1001, ScienCell, Carlsbad, CA, USA) supplemented with 10% fetal bovine serum (FBS, 0025, ScienCell, Carlsbad, CA, USA), 1% endothelial cell growth supplement (ECGS, 1052, ScienCell, Carlsbad, CA, USA), and 1% penicillin-streptomycin (0503, ScienCell, Carlsbad, CA, USA) at 37 °C in a humidified atmosphere of 5% CO_2_. Cells between passages 3 and 6 were used for all experiments.

For the initial concentration-screening experiment, HUVECs were exposed to recombinant human cold-inducible RNA-binding protein (CIRP, APG886Hu01, Cloud-Clone Corp., Wuhan, China) over a concentration range of 10–5000 ng/mL, and cell viability was assessed using the CCK-8 assay. The concentrations used for subsequent dose–response experiments were selected based on this initial screening and previous endothelial-cell studies. For dose–response and time-course studies, HUVECs were treated with eCIRP at 250, 500, or 1000 ng/mL for 12, 24, or 48 h, as indicated. According to the manufacturer’s product information, the protein was generated by prokaryotic expression in *E. coli* and contained an N-terminal His-tag. The purity was >95%, and the endotoxin level was <1.0 EU/μg, as determined by the LAL assay. For inhibitor studies, cells were pre-treated with the selective TLR4 inhibitor TAK-242 (resatorvid; HY-11109, MedChemExpress, Monmouth Junction, NJ, USA)) at 1 μM for 1 h prior to eCIRP (1000 ng/mL) stimulation for 24 h. To evaluate whether TAK-242 itself affected HUVEC viability, cells were treated with vehicle or TAK-242 at the indicated concentrations for 25 h. In parallel, cells were pretreated with vehicle or TAK-242 for 1 h before stimulation with CIRP at 1000 ng/mL for 24 h. Cell viability was then assessed using the CCK-8 assay. For SIRT6 overexpression, HUVECs were transduced with Ad-SIRT6 or Ad-NC (Hanbio, Shanghai, China) at a multiplicity of infection (MOI) of 20 for 48 h, followed by CIRP stimulation. For SIRT6 knockdown, cells were transfected with SIRT6 siRNA (GenePharma, Shanghai, China) or negative control siRNA (si-NC) using TransIT-X2 Dynamic Delivery System (MIR 6010, Dakewe, Shenzhen, China) according to the manufacturer’s instructions. After 48 h transfection, cells were stimulated with CIRP for an additional 24 h.

### 2.4. Cell Viability and LDH Release Assays

Cell viability was assessed using a CCK-8 assay kit (C0038, Beyotime, Shanghai, China) according to the manufacturer’s instructions. HUVECs were seeded in 96-well plates and treated as indicated. After treatment, CCK-8 reagent was added to each well and incubated at 37 °C for 4 h. Absorbance was measured at 450 nm using a microplate reader. Cytotoxicity was evaluated by measuring lactate dehydrogenase (LDH) release in cell culture supernatants using a commercial LDH assay kit (C0017, Beyotime, Shanghai, China) according to the manufacturer’s instructions. LDH release was calculated relative to the corresponding control group.

### 2.5. ELISA

Serum CIRP levels in mice or human were measured using commercial CIRP ELISA kits (CSB-EL005440MO/CSB-EL005440HU, CUSABIO, Wuhan, China) according to the manufacturer’s instructions. The concentration of interleukin-1β (IL-1β) in cell culture supernatants was determined using a human IL-1β ELISA kit (RX106152H, RUIXIN, Quanzhou, China). All samples and standards were run in duplicate. Standard curves were generated to calculate sample concentrations.

### 2.6. Western Blotting

Total protein was extracted from aortic tissues or HUVECs using ice-cold RIPA lysis buffer (P0013B, Beyotime, Shanghai, China) supplemented with 1% protease inhibitor cocktail and 1% phosphatase inhibitor cocktail. Protein concentrations were determined using a BCA protein assay kit (P0010, Beyotime, Shanghai, China). Equal amounts of protein (30–50 μg per lane) were separated by 10% SDS-PAGE and transferred onto polyvinylidene difluoride (PVDF) membranes (Millipore, Burlington, MA, USA). Membranes were blocked with 5% non-fat milk in TBST (Tris-buffered saline containing 0.1% Tween-20) for 2.5 h at room temperature, then incubated overnight at 4 °C with primary antibodies against CIRP (1:500; YN1851, Immunoway, Suzhou, China), SIRT6 (1:1000; ab191385, Abcam, Cambridge, UK), TLR4 (1:1000; YT0744, Immunoway, Suzhou, China), eNOS (1:1000; YT3174, Immunoway, Suzhou, China), β-actin (1:2000; AT0001, Engibody Biotechnology, Dover, DE, USA), p65 (1:2000; CY5034, ABways, Shanghai, China), phospho-p65 (1:500; AP1294, ABclonal, Wuhan, China), TLR2 (1:1000; 66645-1-Ig, Proteintech, Wuhan, China), and TREM-1 (1:500; 11791-1-AP, Proteintech, Wuhan, China). Protein bands were visualized using an enhanced chemiluminescence (ECL) detection system and quantified by densitometry using ImageJ software (version 1.54). The expression levels of CIRP, SIRT6, TLR4, eNOS, TLR2 and TREM-1 were normalized to β-actin. The phosphorylation level of p65 was quantified as the ratio of phospho-p65 to total p65.

### 2.7. SIRT6 Protein Stability Assay

SIRT6 protein stability was evaluated using a cycloheximide (CHX) chase assay. HUVECs were treated with vehicle, CIRP, or CIRP plus TAK-242 as indicated. After the indicated treatments, cells were exposed to 20 μg/mL cycloheximide (CHX; HY-12320, MedChemExpress, Monmouth Junction, NJ, USA) to inhibit de novo protein synthesis. Cells were harvested at 0, 2, 4, 6, and 8 h after CHX treatment. Total protein was extracted and subjected to Western blotting to detect SIRT6 expression. SIRT6 protein levels were normalized to β-actin and then expressed relative to the corresponding 0 h time point in each group.

### 2.8. Quantitative Real-Time PCR

Total RNA was extracted from aortic tissues or HUVECs using TRIzol reagent according to the manufacturer’s protocol. RNA purity and concentration were assessed using a NanoDrop spectrophotometer. Complementary DNA (cDNA) was synthesized from 1 μg of total RNA using the PrimeScript RT Master Mix kit (A241, Genstar, Beijing, China). Quantitative PCR was performed using a CFX96 Real-Time PCR Detection System (Bio-Rad Laboratories, Inc., Hercules, CA, USA) and using Universal SYBR qPCR Master Mix (A301, Genstar, Beijing, China). Relative gene expression was calculated using the 2^−ΔΔCt^ method, with GAPDH as the internal reference gene. The primer sequences used in this study are listed in Table 1. The official gene symbols and corresponding NCBI Gene IDs for the genes mentioned throughout the manuscript are provided in Table 2.

### 2.9. Oxidative Stress

The level of lipid peroxidation was assessed by measuring MDA content using a commercial assay kit (S0131S, Beyotime, Shanghai, China) according to the manufacturer’s protocol. Briefly, serum or cell lysates were mixed with MDA working solution, heated at 100 °C for 30 min, cooled to room temperature, and centrifuged at 1000× *g* for 10 min. The absorbance of the supernatant was measured at 532 nm. GSH-Px activity was determined using a commercial assay kit (S0056, Beyotime, Shanghai, China) based on the rate of glutathione oxidation catalyzed by GSH-Px. The reaction mixture was incubated at room temperature for 15 min, and the absorbance was measured at 340 nm at 1 min intervals for 5 min. Catalase (CAT) activity was measured using a commercial assay kit (A007-1-1, Nanjing Jiancheng Bioengineering Institute, Nanjing, China) according to the manufacturer’s instructions. The reaction was terminated by adding ammonium molybdate, and the residual hydrogen peroxide formed a yellow complex with ammonium molybdate. The absorbance was measured at 405 nm, and CAT activity was calculated according to the manufacturer’s formula. Intracellular reactive oxygen species (ROS) levels in HUVECs were measured using the fluorescent probe 2′,7′-dichlorofluorescein diacetate (DCFH-DA; S0033S, Beyotime, Shanghai, China). Cells were incubated with 10 μM DCFH-DA in serum-free medium for 30 min at 37 °C in the dark. After two washes with PBS, fluorescence images were captured using a fluorescence microscope at an excitation wavelength of 488 nm. Fluorescence intensity was quantified using ImageJ software (version 1.54).

### 2.10. Nitric Oxide (NO) and eNOS Activity Assays

Total nitric oxide production was indirectly quantified by measuring nitrite levels in serum or cell lysates using the Griess reagent assay (S0021S, Beyotime, Shanghai, China). Absorbance was measured at 540 nm. eNOS activity was determined using a commercial eNOS activity assay kit (A014-1-2, Nanjing Jiancheng Bioengineering Institute, Nanjing, China) based on the conversion of L-arginine to L-citrulline, following the manufacturer’s instructions.

### 2.11. THP-1 Cell Adhesion Assay

THP-1 cell adhesion to endothelial cells was assessed using a calcein-AM labeling assay. Briefly, THP-1 monocytes were labeled with 2 μM calcein-AM (C2012; Beyotime, Shanghai, China) for 30 min at 37 °C in the dark. After labeling, the cells were washed three times with PBS to remove excess fluorescent dye and then resuspended in culture medium. Calcein-AM-labeled THP-1 cells were added to the treated HUVEC monolayers at a density of 5 × 10^3^ cells per well and co-incubated for 1 h at 37 °C. Non-adherent THP-1 cells were removed by gently washing the wells three times with PBS. Fluorescence images and corresponding bright-field images were captured using a fluorescence microscope. The number of adherent THP-1 cells was quantified from fluorescence images using ImageJ software (version 1.54).

### 2.12. Immunoprecipitation

Immunoprecipitation assays were performed to assess eNOS acetylation and the interaction between SIRT6 and eNOS. Briefly, HUVECs were lysed in IP lysis buffer supplemented with 1% protease inhibitor cocktail. Cell lysates were collected after centrifugation, and a portion of each lysate was retained as input control. Protein A/G magnetic beads (HY-K0202; MedChemExpress, Monmouth Junction, NJ, USA) were washed twice with washing buffer and then incubated with anti-SIRT6 antibody, anti-eNOS antibody, anti-acetylated-lysine antibody, or control IgG (98136-1-RR; Proteintech, Wuhan, China) at 4 °C for 2 h with rotation. The prepared cell lysates were then added to the antibody-coupled beads and incubated overnight at 4 °C with rotation. After incubation, the beads were washed four times with binding/washing buffer. Bound proteins were eluted by boiling the beads in 1× SDS-PAGE loading buffer at 95 °C for 5 min. The eluates were collected by magnetic separation and subjected to Western blotting. For eNOS acetylation analysis, acetylated proteins were immunoprecipitated using an anti-acetylated-lysine antibody, followed by immunoblotting for eNOS. For co-immunoprecipitation analysis, SIRT6 was immunoprecipitated followed by immunoblotting for eNOS, and reciprocal immunoprecipitation was performed by immunoprecipitating eNOS followed by immunoblotting for SIRT6. IgG was used as a negative control.

### 2.13. Immunofluorescence

Frozen aortic root tissues were cut into 7 μm thick cryosections and rewarmed at room temperature for 30 min, fixed in 4% paraformaldehyde solution, permeabilized with 0.3% Triton X-100, blocked with goat serum for 1 h at room temperature. The sections were incubated with anti-TLR4 (1:50, YT0744, Immunoway, Suzhou, China) and anti-CD31 (1:500, GB12063, Servicebio, Wuhan, China) overnight at 4 °C. Then, samples were incubated with secondary antibodies (goat anti-rabbit IgG (H&L)-Alexa Fluor 594, goat anti-mouse IgG (H&L)-Alexa Fluor 488) for 1 h at room temperature. Nuclei were stained with DAPI. After routine immunofluorescence staining, images were captured using a confocal laser scanning microscope (Olympus FV3000, Olympus, Tokyo, Japan).

### 2.14. Bioinformatics Analysis

Differential expression analysis was performed using a publicly available CIRP-knockdown RNA-seq dataset (GSE175747) and an in-house TNF-α-stimulated HUVEC transcriptomic dataset. The GSE175747 dataset was used as a CIRP-perturbation dataset, whereas the TNF-α-stimulated HUVEC dataset was used as an endothelial inflammatory injury-related transcriptomic signature. Because the two datasets were derived from different biological contexts, the analysis was performed as an exploratory intersection analysis rather than as a direct identification of CIRP-regulated endothelial target genes. For the in-house HUVEC dataset, HUVECs were treated with TNF-α (40 ng/mL) for 12 h, and RNA-seq was performed with three independent biological replicates per group. Data are available from the corresponding author upon reasonable request. Differentially expressed genes were identified using DESeq2 (version 1.42.1), with thresholds of |log2 fold change| > 0.5 and *p* < 0.05. Venn diagram analysis was performed to identify overlapping differentially expressed genes between the two datasets. A total of 56 overlapping genes were identified, and the complete gene list is provided in Supplementary Table S5. Gene Ontology (GO) biological process enrichment analysis and Kyoto Encyclopedia of Genes and Genomes (KEGG) pathway enrichment analysis of the overlapping genes were performed using the clusterProfiler package (version 4.10.1) in R (version 4.3.2). 

### 2.15. Statistical Analysis

All data are presented as mean ± standard error of the mean (SEM). Statistical analyses were performed using GraphPad Prism 8.0 software. For animal experiments, *n* represents the number of mice. For clinical analyses, *n* represents the number of participants or patients. For cell-based experiments, *n* represents independent biological replicates performed on separate occasions using independently cultured cells. Technical replicates within each experiment were averaged and were not treated as independent observations for statistical analysis. Normality was assessed using the Shapiro–Wilk test. For comparisons between two groups, an unpaired Student’s *t*-test was used for normally distributed data, whereas the Mann–Whitney U test was used for non-normally distributed data. For comparisons among three or more groups, one-way analysis of variance (ANOVA) followed by Tukey’s multiple comparisons test was used. SIRT6 protein degradation curves in the cycloheximide chase assay were analyzed using two-way ANOVA followed by multiple comparisons tests. Correlation analyses between serum CIRP levels and clinical parameters were performed using Pearson’s correlation for normally distributed variables and Spearman’s rank correlation for non-normally distributed variables. Categorical variables were compared using the chi-square test or Fisher’s exact test, as appropriate. All tests were two-tailed, and *p* < 0.05 was considered statistically significant. Significance levels were indicated as * *p* < 0.05 and ** *p* < 0.01; ns indicates not significant.

## 3. Results

### 3.1. HFD-Fed ApoE^−/−^ Mice Exhibit Elevated Circulating CIRP, Redox Imbalance, Endothelial Dysfunction, and Vascular Inflammation

**Figure 1:**
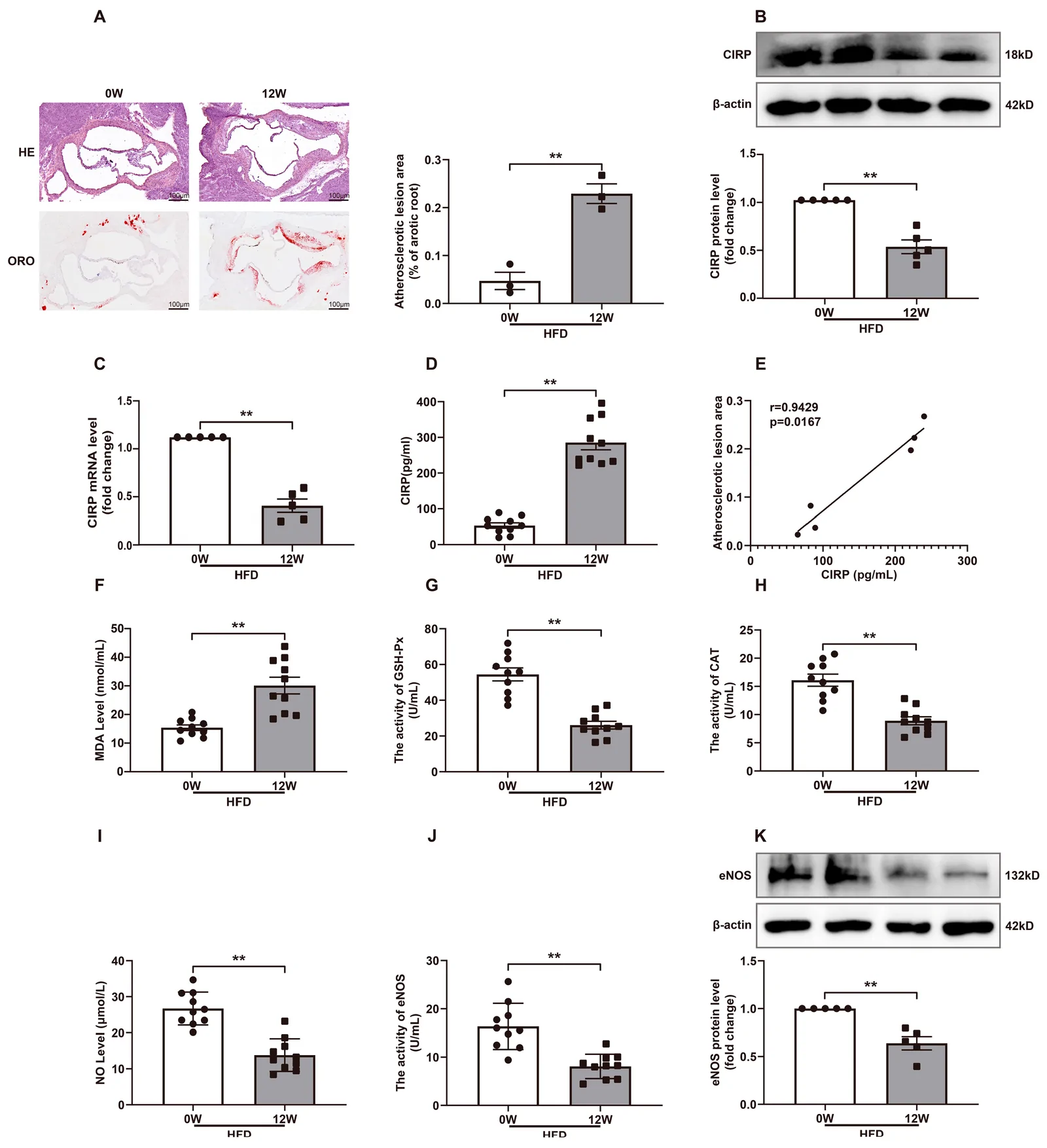
HFD-fed ApoE^−/−^ mice exhibit elevated circulating CIRP, systemic redox imbalance, and endothelial dysfunction. (**A**) Representative images of H&E and Oil Red O staining in aortic root sections from ApoE^−/−^ mice, along with quantification data, *n* = 3. (**B**,**C**) CIRP expression in the aorta was determined by Western blotting and RT-qPCR, *n* = 5. (**D**) Serum CIRP levels measured by ELISA, *n* = 10. (**E**) Correlation between serum CIRP levels and atherosclerotic plaque area, *n* = 6. (**F**–**H**) Serum MDA levels, GSH-Px and CAT activity were measured using commercial assay kits, *n* = 10. (**I**,**J**) Serum NO levels and eNOS activity were measured using assay kits, *n* = 10. (**K**) The expression of eNOS in the aorta was determined by Western blotting, *n* = 5. Data are presented as mean ± SEM. Dots represent individual mice. Differences between two groups were analyzed using an unpaired Student’s *t*-test. Correlation analyses were conducted using Pearson’s correlation coefficient. ** *p* < 0.01.

To characterize CIRP-related changes under atherosclerosis-associated condition, ApoE^−/−^ mice were examined at 0 w and after 12 weeks of high-fat diet (HFD) feeding. Histological analysis of aortic roots revealed significant atherosclerotic lesions at 12 weeks compared to 0 weeks, as evidenced by H&E and Oil Red O staining (Figure 1A). We next assessed CIRP expression in aortic tissues and serum. Western blotting and RT-qPCR analyses showed that CIRP protein and mRNA levels were reduced in aortic tissues from HFD-fed ApoE^−/−^ mice (Figure 1B,C). In contrast, serum CIRP levels were significantly elevated after 12 weeks of HFD feeding (Figure 1D). Moreover, serum CIRP levels were positively correlated with atherosclerotic lesion area (Figure 1E). In a preliminary time-course analysis, serum CIRP levels increased during HFD feeding at 0, 4, 8, and 12 weeks, with higher levels observed at later time points (Figure S1D). HFD-fed ApoE^−/−^ mice also exhibited systemic redox imbalance. Serum malondialdehyde (MDA) levels were significantly increased (Figure 1F), whereas glutathione peroxidase (GSH-Px) and catalase (CAT) activities were decreased after HFD feeding (Figure 1G,H). In addition, HFD feeding was accompanied by endothelial dysfunction, as indicated by reduced serum nitric oxide (NO) levels, decreased eNOS activity, and lower aortic eNOS protein expression (Figure 1I–K). Consistent with vascular inflammation, aortic mRNA levels of TNF-α, IL-1β and IL-6 were significantly increased in HFD-fed ApoE^−/−^ mice compared with baseline mice (Figure S1A–C). Collectively, these findings show that HFD-fed ApoE^−/−^ mice exhibit increased circulating CIRP, systemic redox imbalance, endothelial dysfunction, and vascular inflammation. These findings indicate that elevated serum CIRP occurs in parallel with atherosclerosis-related vascular injury in HFD-fed ApoE^−/−^ mice.

### 3.2. CIRP Induces Redox-Inflammatory Endothelial Injury in HUVECs

**Figure 2:**
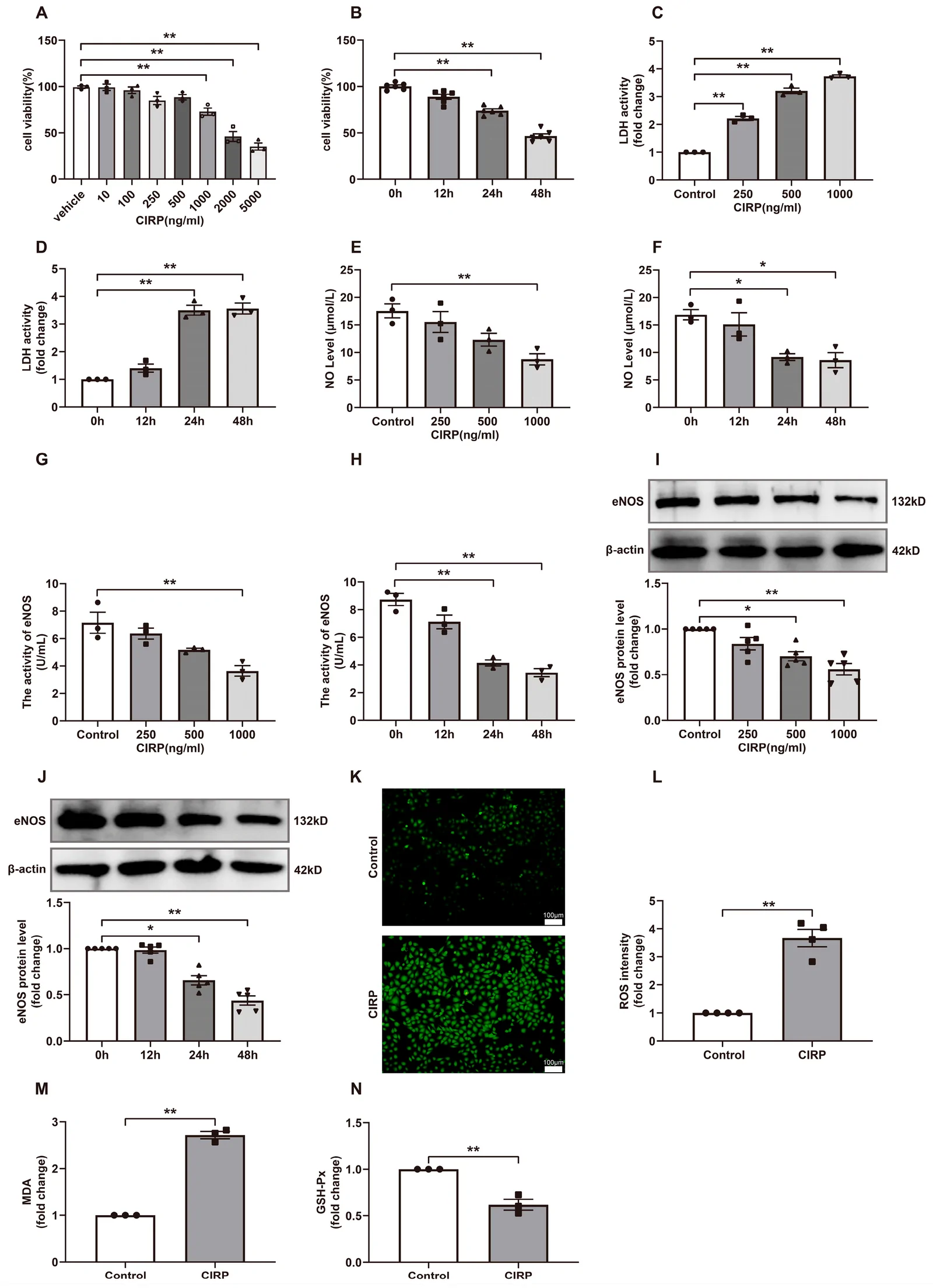
CIRP induces endothelial dysfunction and oxidative stress in HUVECs. (**A**) Cell viability of HUVECs after treatment with different concentrations of CIRP was assessed by CCK-8 assay, *n* = 3. (**B**) Time-course analysis of cell viability in HUVECs treated with 1000 ng/mL CIRP for 0, 12, 24, and 48 h, *n* = 3. (**C**) LDH release in HUVECs treated with different concentrations of CIRP, *n* = 3. (**D**) Time-course analysis of LDH release after 1000 ng/mL CIRP treatment, *n* = 3. (**E**,**F**) NO production was measured after dose- and time-dependent CIRP treatment, *n* = 3. (**G**,**H**) eNOS activity was determined after dose- and time-dependent CIRP treatment, *n* = 3. (**I**,**J**) Dose- and time-dependent changes in eNOS protein expression, *n* = 5. (**K**) Representative images of intracellular ROS detected by DCFH-DA fluorescence microscopy. (**L**) Fluorescence intensity was measured using ImageJ, *n* = 4. (**M**,**N**) Quantification of MDA levels (**M**) and GSH-Px activity (**N**) in HUVECs after CIRP treatment, *n* = 3. Data are presented as mean ± SEM. Dots represent independent biological replicates. Differences between two groups were analyzed by an unpaired Student’s *t*-test. Comparisons among multiple groups were performed by one-way ANOVA. * *p* < 0.05, ** *p* < 0.01.

To investigate the effects of extracellular CIRP on endothelial injury, HUVECs were treated with recombinant CIRP at different concentrations and time points. CIRP reduced HUVEC viability and increased LDH release in a dose- and time-dependent manner (Figure 2A–D). Because 1000 ng/mL CIRP induced consistent endothelial injury-related responses, this concentration was selected for subsequent mechanistic experiments. We next assessed endothelial functional status. CIRP treatment decreased NO production in both concentration- and time-dependent manners (Figure 2E,F). Similarly, eNOS activity was reduced after CIRP stimulation (Figure 2G,H). Western blotting further showed that CIRP decreased eNOS protein expression in HUVECs in a dose- and time-dependent manner (Figure 2I,J), suggesting impairment of endothelial NO/eNOS signaling. Consistent with the redox alterations observed in vivo, CIRP increased intracellular reactive oxygen species (ROS) generation, as shown by DCFH-DA fluorescence imaging and fluorescence intensity quantification (Figure 2K,L). In addition, CIRP increased MDA levels and decreased GSH-Px activity in HUVECs (Figure 2M,N), indicating disruption of cellular redox homeostasis. CIRP also induced inflammatory responses in HUVECs. IL-1β secretion was increased after CIRP treatment in a dose- and time-dependent manner (Figure S2A,B). In parallel, TNF-α and IL-1β mRNA levels were upregulated after CIRP stimulation (Figure S2C–F). Together, these findings show that recombinant CIRP exposure induces endothelial cytotoxicity, NO/eNOS dysfunction, redox imbalance and inflammatory responses in HUVECs.

### 3.3. SIRT6 Restoration Attenuates CIRP-Induced Endothelial Dysfunction and Redox Imbalance

**Figure 3:**
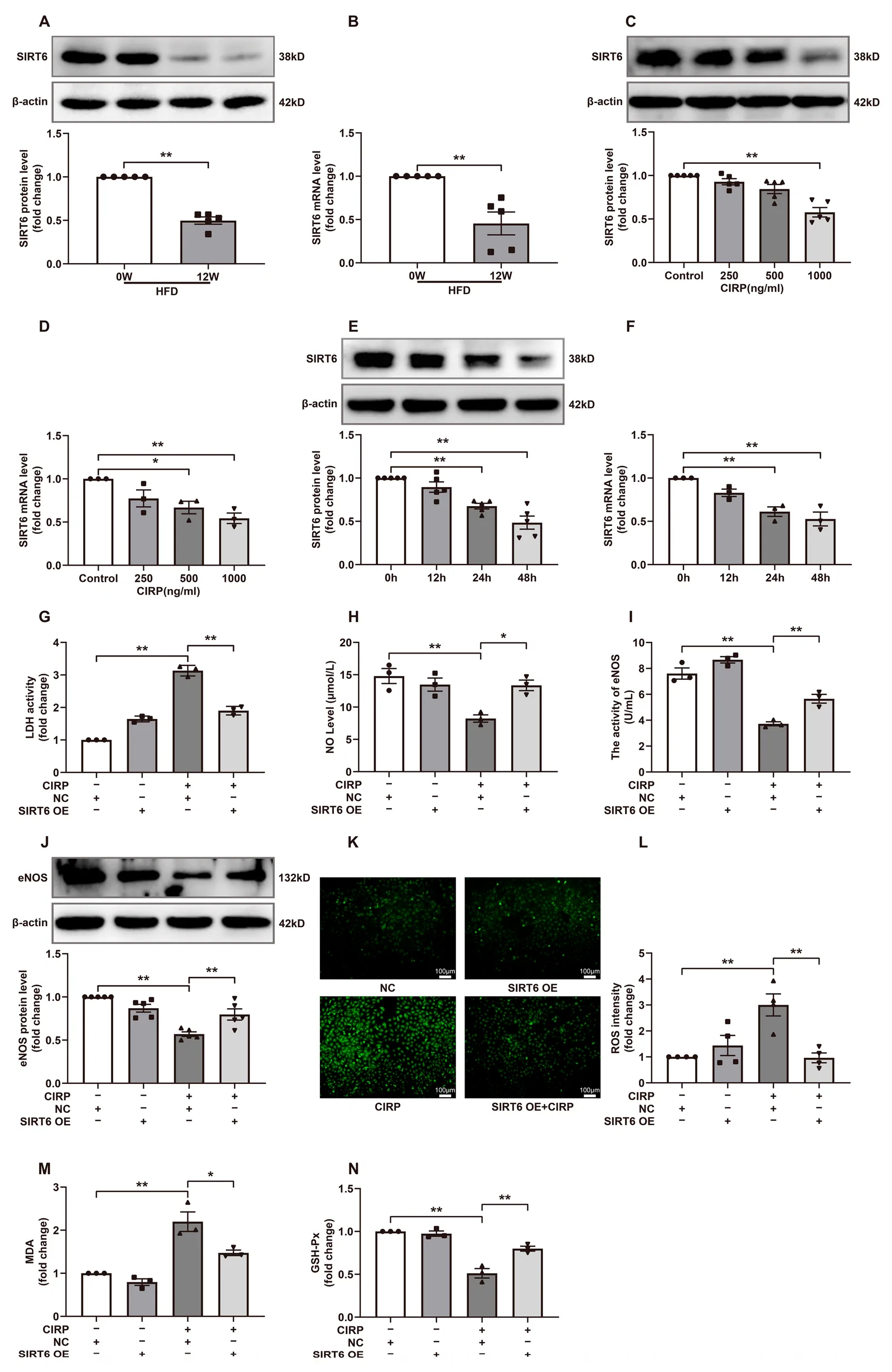
SIRT6 overexpression attenuates CIRP-induced endothelial dysfunction and oxidative stress. (**A**,**B**) SIRT6 expression in aortas from HFD-fed ApoE^−/−^ mice was determined by Western blotting and RT-qPCR, *n* = 5. (**C**,**D**) Dose-dependent effects of CIRP on SIRT6 protein and mRNA expression, *n* = 3. (**E**,**F**) The expression of SIRT6 in HUVECs was assessed at the indicated time points (0, 12, 24, and 48 h) following treatment with 1000 ng/mL CIRP, *n* = 3. (**G**–**I**) Effects of SIRT6 overexpression on CIRP-induced LDH release, NO production, and eNOS activity, *n* = 3. (**J**) eNOS protein expression after SIRT6 overexpression was determined by Western blotting, *n* = 5. (**K**,**L**) Intracellular ROS generation was detected after SIRT6 overexpression, *n* = 4. (**M**,**N**) MDA levels and GSH-Px activity were measured using commercial assay kits, *n* = 3. Data are presented as mean ± SEM. Dots indicate individual mice or independent biological replicates. Differences between two groups were analyzed by an unpaired Student’s *t*-test. Comparisons among multiple groups were performed by one-way ANOVA. * *p* < 0.05, ** *p* < 0.01.

Given the important role of SIRT6 in endothelial homeostasis, we next examined whether SIRT6 was involved in CIRP-induced endothelial injury. In ApoE^−/−^ mice, SIRT6 protein and mRNA levels were significantly reduced in aortic tissues after 12 weeks of HFD feeding (Figure 3A,B). Consistently, CIRP treatment also decreased SIRT6 expression in a concentration- and time-dependent manner in HUVECs (Figure 3C–F). We then restored SIRT6 expression by adenoviral overexpression. The overexpression efficiency was confirmed by Western blotting and RT-qPCR (Figure S3A,B). SIRT6 overexpression attenuated CIRP-induced cytotoxicity (Figure 3G). It also partially restored NO production (Figure 3H), improved eNOS activity (Figure 3I), and partially recovered eNOS protein expression (Figure 3J), suggesting improved endothelial NO/eNOS signaling. We next examined whether SIRT6 overexpression mitigates CIRP-induced redox imbalance. Indeed, SIRT6 restoration reduced ROS accumulation (Figure 3K,L), attenuated MDA levels (Figure 3M), and restored GSH-Px activity (Figure 3N). Concurrently, SIRT6 restoration also attenuated CIRP-induced inflammatory responses, as reflected by reduced IL-1β secretion and decreased TNF-α and IL-1β mRNA expression (Figure S3C–E). As a complementary approach, we also examined the effect of SIRT6 knockdown under CIRP stimulation. The efficiency of SIRT6 silencing was confirmed by Western blotting and RT-qPCR (Figure S6A,B). Under basal conditions, SIRT6 knockdown increased several injury-related readouts. Under CIRP stimulation, additional SIRT6 knockdown further increased LDH release and IL-1β secretion. However, it did not significantly further alter NO production, eNOS activity, eNOS protein expression, or TNF-α and IL-1β mRNA levels compared with CIRP plus control siRNA (Figure S6C–I). This endpoint-specific pattern suggests that additional SIRT6 silencing selectively aggravated some CIRP-induced responses, whereas the absence of further changes in other parameters may reflect floor or ceiling effects, limited assay dynamic range, or pathway saturation under the present experimental conditions. Together, these findings indicate that SIRT6 is downregulated under CIRP stimulation. Restoration of SIRT6 partially attenuates CIRP-induced endothelial dysfunction, redox imbalance, and inflammatory responses in HUVECs. These results suggest that SIRT6 contributes to CIRP-induced endothelial injury but is unlikely to be the only regulatory factor.

### 3.4. Pharmacological TLR4 Inhibition Attenuates CIRP-Associated Endothelial Injury and SIRT6 Downregulation

**Figure 4:**
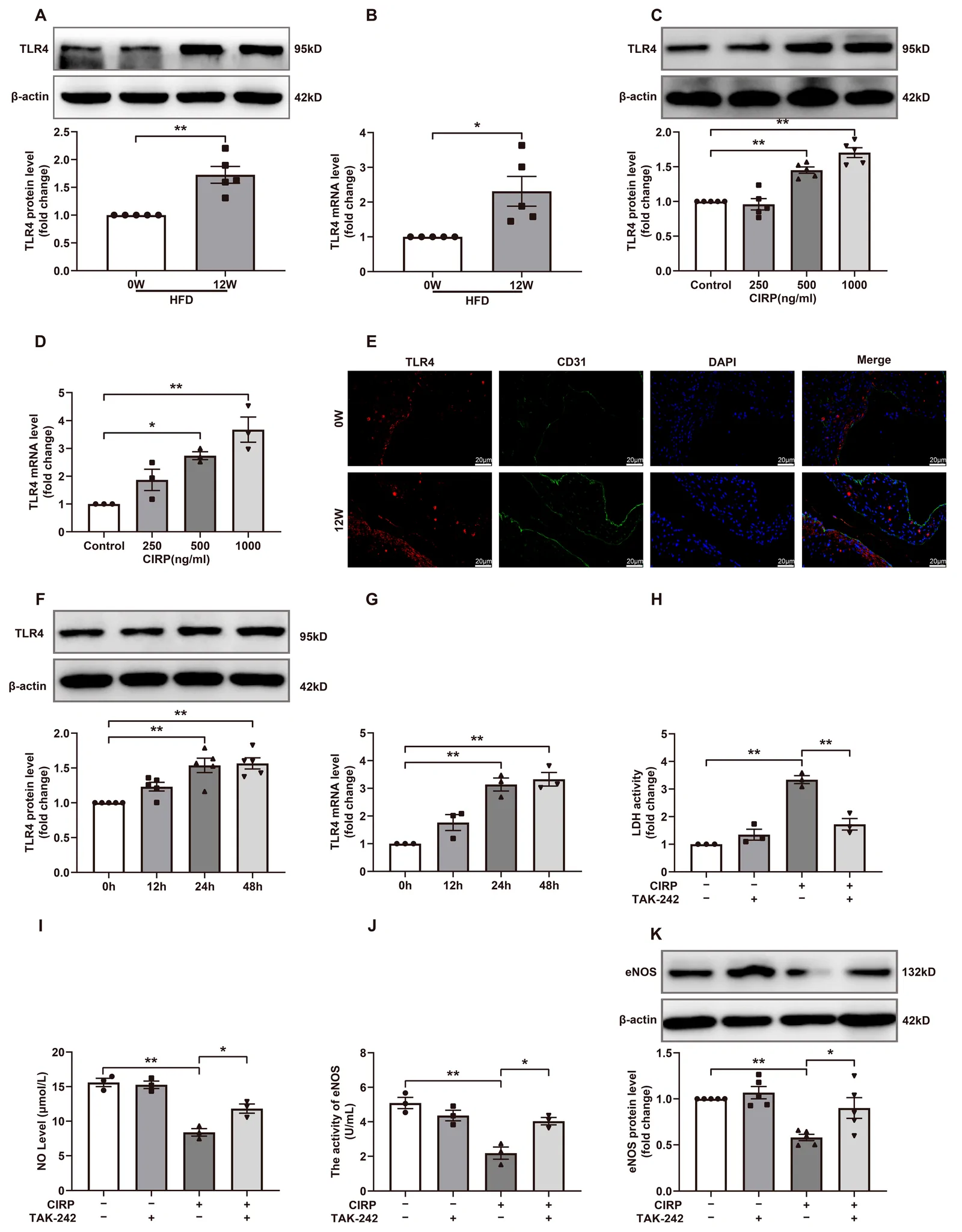
TLR4 inhibition attenuates CIRP-induced endothelial dysfunction. (**A**,**B**) TLR4 expression in aortas from HFD-fed ApoE^−/−^ mice was analyzed by Western blotting and RT-qPCR, *n* = 5. (**C**,**D**) The protein and mRNA levels of TLR4 in HUVECs were assessed after treatment with 250, 500, and 1000 ng/mL CIRP, *n* = 3. (**E**) Representative immunofluorescence staining images of TLR4 (red), CD31 (green), and DAPI (blue) in aortic root sections. (**F**,**G**) Time-dependent effects of CIRP on TLR4 protein and mRNA expression, *n* = 3. HUVECs were pretreated with TAK-242 for 1 h and then were incubated with CIRP for 24 h. (**H**) Effects of TAK-242 on CIRP-induced LDH release, *n* = 3. (**I**,**J**) Effects of TAK-242 on NO production and eNOS activity after CIRP stimulation, *n* = 3. (**K**) The expression of eNOS in HUVECs was determined by Western blotting, *n* = 5. Data are presented as mean ± SEM. Dots indicate individual mice or independent biological replicates. Differences between two groups were analyzed by an unpaired Student’s *t*-test. Comparisons among multiple groups were performed by one-way ANOVA. * *p* < 0.05, ** *p* < 0.01.

Because extracellular CIRP has been reported to interact with TLR4, we next examined the effects of pharmacological TLR4 inhibition on CIRP-associated endothelial responses and SIRT6 downregulation. In HFD-fed ApoE^−/−^ mice, aortic TLR4 expression was increased at both protein and mRNA levels (Figure 4A,B). CIRP stimulation also increased TLR4 protein and mRNA expression in HUVECs in concentration-dependent manners (Figure 4C,D). Immunofluorescence staining also showed increased TLR4 signal in CD31-positive endothelial cells within aortic root lesions from HFD-fed ApoE^−/−^ mice (Figure 4E). In addition, CIRP increased TLR4 protein and mRNA expression in HUVECs over time (Figure 4F,G). To assess the potential contribution of TLR4, HUVECs were pretreated with TAK-242, a pharmacological TLR4 inhibitor, before CIRP stimulation. We first evaluated whether TAK-242 affected HUVEC viability under the present experimental conditions. TAK-242 at the concentration used in this study (1 μM) did not significantly reduce HUVEC viability when administered alone. Under CIRP-stimulated conditions, TAK-242 partially restored cell viability without causing additional cytotoxicity at the working concentration (Figure S4H,I). TAK-242 reduced CIRP-induced LDH release (Figure 4H). It also restored NO production, improved eNOS activity, and partially recovered eNOS protein expression in CIRP-treated HUVECs (Figure 4I–K), suggesting attenuation of CIRP-induced endothelial dysfunction. TAK-242 also attenuated inflammatory responses. IL-1β secretion was decreased after TAK-242 treatment, accompanied by reduced TNF-α and IL-1β mRNA expression (Figure S4A–C). We further examined the relationship between TLR4 inhibition and SIRT6 expression. Notably, TAK-242 treatment suppressed CIRP-induced reduction in SIRT6 protein and mRNA levels (Figure S4D,E). In the cycloheximide chase assay, CIRP accelerated the decline of SIRT6 protein after inhibition of de novo protein synthesis, whereas TAK-242 partially preserved SIRT6 protein levels over time (Figure S4F,G). These findings show that TAK-242 treatment was associated with attenuation of CIRP-induced SIRT6 downregulation and partial preservation of SIRT6 protein stability under the present experimental conditions. To examine the possibility of reciprocal regulation, we assessed TLR4 expression following SIRT6 overexpression. SIRT6 overexpression did not significantly alter either basal or CIRP-induced TLR4 mRNA or total protein expression (Figure S3F,G).

Because extracellular CIRP may also act through receptors other than TLR4, we further evaluated several reported CIRP-associated receptors in HUVECs. CIRP did not significantly alter TLR2 protein or mRNA expression under either concentration- or time-dependent stimulation conditions (Figure S5A–D). We also examined TREM-1 expression. TREM-1 protein was expressed at very low abundance in HUVECs and was only weakly detectable. Therefore, these protein data were interpreted cautiously. TREM-1 expression was not consistently increased in response to different CIRP concentrations, although a modest late increase in TREM-1 protein was observed after prolonged CIRP exposure (Figure S5E–H). In addition, CIRP increased IL6R mRNA expression under high-dose and prolonged stimulation conditions (Figure S5I,J). AGER mRNA also showed an increasing trend after CIRP treatment, with a more evident increase after prolonged stimulation (Figure S5K,L). Collectively, pharmacological inhibition with TAK-242 partially attenuated CIRP-associated endothelial dysfunction, inflammatory responses, and SIRT6 downregulation in HUVECs. The receptor-expression analysis further indicates that other CIRP-associated receptors may also be altered by CIRP exposure; therefore, TLR4-independent signaling cannot be excluded.

### 3.5. SIRT6 Partially Contributes to the Protective Effects of TLR4 Inhibition Against CIRP-Induced Endothelial Injury

**Figure 5:**
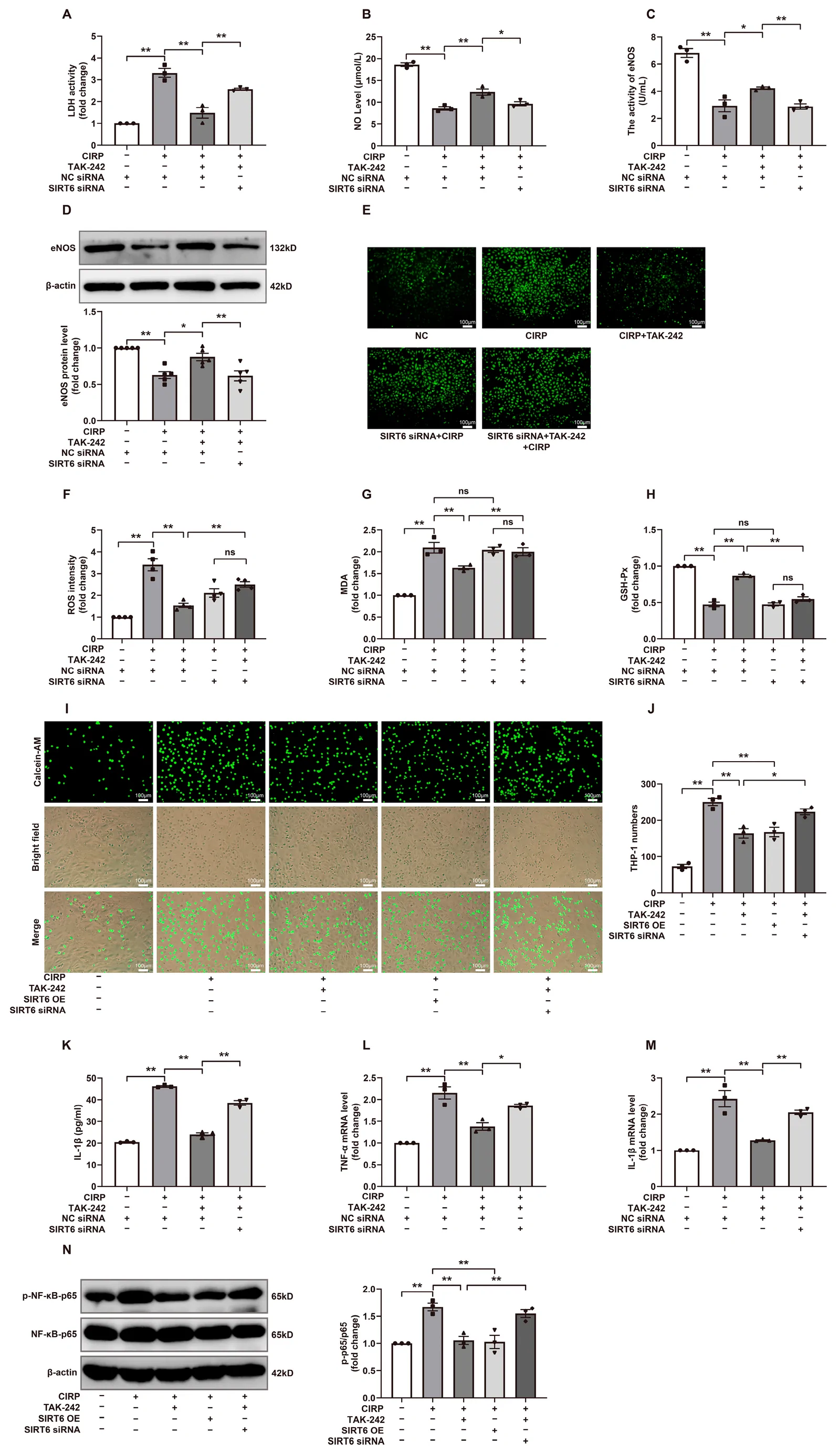
SIRT6 contributes to the protective effects of TLR4 inhibition against CIRP-induced endothelial injury. HUVECs were treated with CIRP in the presence or absence of TAK-242, SIRT6 overexpression, or SIRT6 siRNA as indicated. Cells were pretreated with TAK-242 for 1 h before CIRP stimulation and then incubated with CIRP for 24 h. (**A**) LDH release was measured to assess cytotoxicity, *n* = 3. (**B**,**C**) NO production and eNOS activity were measured using assay kits, *n* = 3. (**D**) eNOS protein expression in HUVECs was determined by Western blotting, *n* = 5. (**E**,**F**) Intracellular ROS levels were detected using DCFH-DA fluorescence microscopy. Fluorescence intensity was measured using ImageJ, *n* = 4. (**G**,**H**) MDA levels and GSH-Px activity were measured using commercial assay kits, *n* = 3. (**I**,**J**) Representative fluorescence and bright-field images showing the adhesion of calcein-AM-labeled THP-1 cells to endothelial cells. The number of calcein-AM-positive adherent THP-1 cells was quantified using ImageJ, *n* = 3. (**K**) IL-1β secretion in culture supernatants was measured by ELISA, *n* = 3. (**L**,**M**) TNF-α and IL-1β mRNA expression was analyzed by RT-qPCR, *n* = 3. (**N**) p-p65 and total p65 protein expression were determined by Western blotting, and the p-p65/p65 ratio was quantified, *n* = 3. Data are presented as mean ± SEM. Dots indicate independent biological replicates. Data were analyzed by one-way ANOVA. * *p* < 0.05, ** *p* < 0.01; ns, not significant.

To further examine the functional relationship between pharmacological TLR4 inhibition and SIRT6 in CIRP-induced endothelial injury, HUVECs were treated with TAK-242 in the presence or absence of SIRT6 siRNA under CIRP stimulation. While TAK-242 treatment alone attenuated CIRP-induced cytotoxicity, SIRT6 knockdown reduced this protective effect, as indicated by increased LDH release (Figure 5A). TAK-242 also restored NO production, improved eNOS activity, and partially recovered eNOS protein expression in CIRP-treated HUVECs (Figure 5B–D). These protective effects were reduced after SIRT6 silencing, suggesting that SIRT6 contributes to the preservation of endothelial NO/eNOS signaling during pharmacological TLR4 inhibition. We next examined oxidative stress-related changes. TAK-242 reduced CIRP-induced ROS accumulation and MDA levels and restored GSH-Px activity in CIRP-treated HUVECs (Figure 5E–H). However, SIRT6 silencing significantly weakened these effects of TAK-242. In addition, ROS accumulation, MDA levels, and GSH-Px activity in the CIRP + TAK-242 + siSIRT6 group were not significantly different from those in the CIRP + siSIRT6 group, supporting a partial contribution of SIRT6 to the antioxidant effects associated with TAK-242 treatment. We further evaluated monocyte adhesion and inflammatory responses. CIRP increased the adhesion of calcein-AM-labeled THP-1 cells to HUVECs, whereas TAK-242 treatment reduced THP-1 adhesion (Figure 5I,J). SIRT6 overexpression also attenuated CIRP-induced THP-1 adhesion. Conversely, SIRT6 silencing partially reversed the inhibitory effect of TAK-242 on THP-1 adhesion. Consistently, TAK-242 reduced IL-1β secretion and decreased TNF-α and IL-1β mRNA expression in CIRP-stimulated HUVECs, whereas SIRT6 silencing weakened these anti-inflammatory effects (Figure 5K–M). Finally, NF-κB activation was assessed by detecting p65 phosphorylation. CIRP increased the p-p65/p65 ratio in HUVECs, whereas TAK-242 treatment reduced p65 phosphorylation (Figure 5N). SIRT6 overexpression also decreased CIRP-induced p65 phosphorylation. In contrast, SIRT6 silencing partially restored p65 phosphorylation under TAK-242 treatment, suggesting that SIRT6 contributes to the inhibitory effect of TAK-242 on CIRP-induced NF-κB activation. Collectively, these findings support a partial contribution of SIRT6 to the protective effects associated with TAK-242 treatment against CIRP-induced endothelial dysfunction, oxidative stress, inflammatory responses, monocyte adhesion, and NF-κB activation. The incomplete reversal after SIRT6 silencing further indicates that additional SIRT6-independent mechanisms are likely involved.

### 3.6. CIRP-Induced Endothelial Dysfunction Is Accompanied by Altered eNOS Acetylation

**Figure 6:**
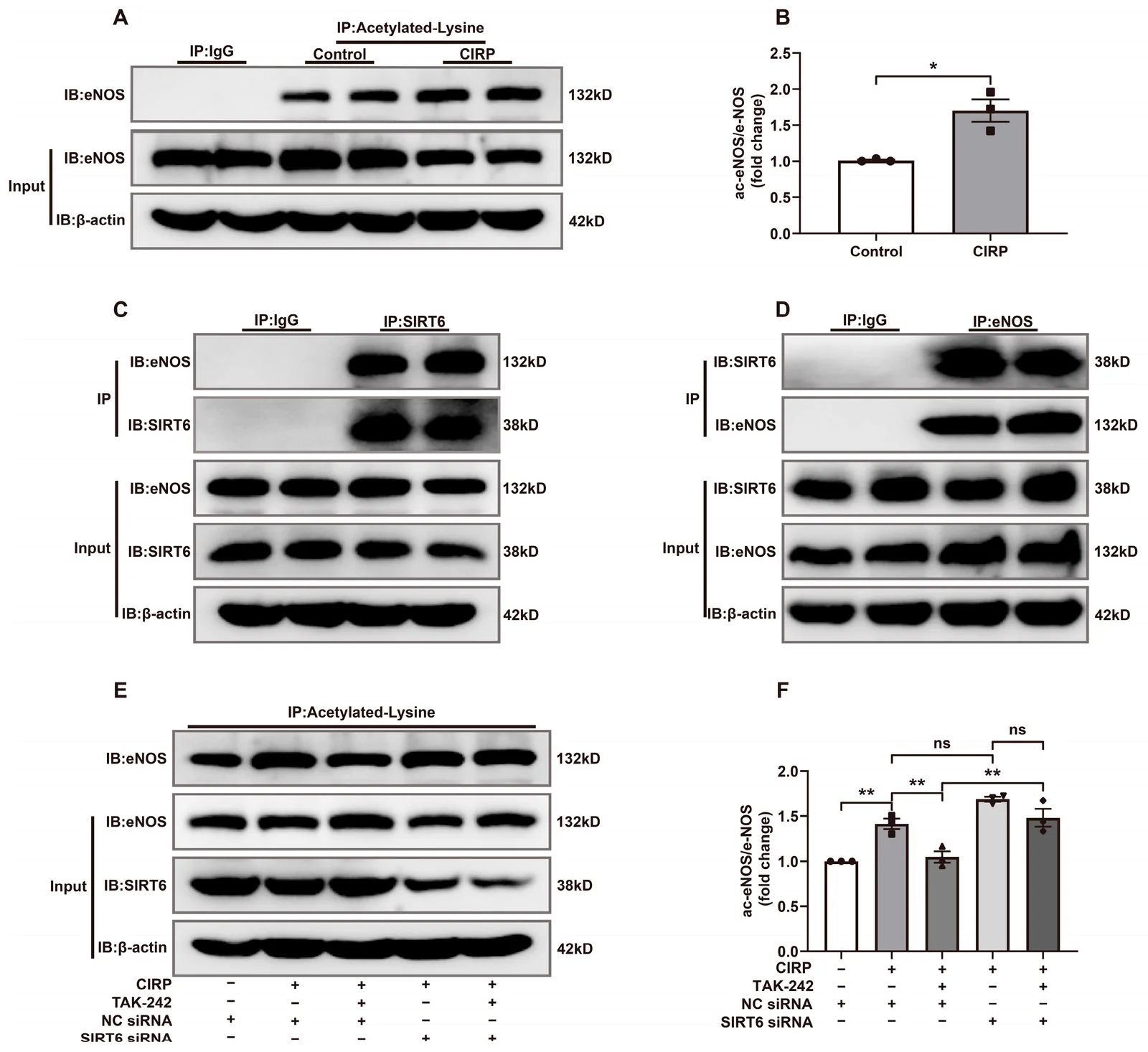
CIRP-induced eNOS acetylation is associated with TLR4 inhibition and SIRT6 expression status. (**A**,**B**) HUVECs were treated with CIRP, and eNOS acetylation was examined by immunoprecipitation using an anti-acetylated-lysine antibody followed by immunoblotting for eNOS. Representative blots are shown in (**A**), and quantification of acetylated eNOS relative to total eNOS is shown in (**B**). (**C**) Co-immunoprecipitation was performed to assess the association between SIRT6 and eNOS in HUVECs. SIRT6 was immunoprecipitated and eNOS was detected by immunoblotting. IgG served as a negative control. (**D**) Reciprocal co-immunoprecipitation was performed by immunoprecipitating eNOS followed by immunoblotting for SIRT6. Corresponding input controls are shown to indicate protein expression in total cell lysates. (**E**,**F**) HUVECs were treated with CIRP in the presence or absence of TAK-242 and/or SIRT6 siRNA. eNOS acetylation was assessed by immunoprecipitation using an anti-acetylated-lysine antibody followed by immunoblotting for eNOS. Data are presented as mean ± SEM, *n* = 3. Dots indicate independent biological replicates. Differences between two groups were analyzed by an unpaired Student’s *t*-test. Comparisons among multiple groups were performed by one-way ANOVA. * *p* < 0.05, ** *p* < 0.01; ns, not significant.

As shown above, CIRP reduced NO production and eNOS activity in HUVECs. However, changes in eNOS function may not be fully explained by altered eNOS protein expression alone, because eNOS activity can also be regulated by post-translational modifications, including acetylation. We therefore examined whether CIRP stimulation was associated with changes in eNOS acetylation. Immunoprecipitation using an anti-acetylated-lysine antibody followed by immunoblotting for eNOS showed that CIRP treatment increased overall eNOS acetylation in HUVECs (Figure 6A,B). Input controls for eNOS and β-actin were included to show protein expression in total cell lysates. We next examined the association between SIRT6 and eNOS in this model. Co-immunoprecipitation analysis showed that eNOS was detected in SIRT6 immunoprecipitates, with IgG serving as a negative control (Figure 6C). Reciprocal co-immunoprecipitation further showed that SIRT6 was detected in eNOS immunoprecipitates (Figure 6D). Corresponding input controls were included to confirm SIRT6 and eNOS expression in total cell lysates. We further evaluated whether TLR4 inhibition and SIRT6 silencing affected CIRP-induced changes in eNOS acetylation. TAK-242 attenuated the increase in eNOS acetylation induced by CIRP. In the presence of SIRT6 silencing, the inhibitory effect of TAK-242 on eNOS acetylation was weakened (Figure 6E,F). Together, these findings indicate that CIRP-induced endothelial dysfunction is accompanied by increased overall eNOS acetylation. The changes in eNOS acetylation were associated with TAK-242 treatment and SIRT6 expression status, suggesting a potential link between TLR4 inhibition, SIRT6 status, and eNOS acetylation. However, these experiments assessed overall eNOS acetylation, and the specific acetylation sites were not identified.

### 3.7. Exploratory Transcriptomic Analysis Support CIRP-Associated Inflammatory and Oxidative Stress Signatures

**Figure 7:**
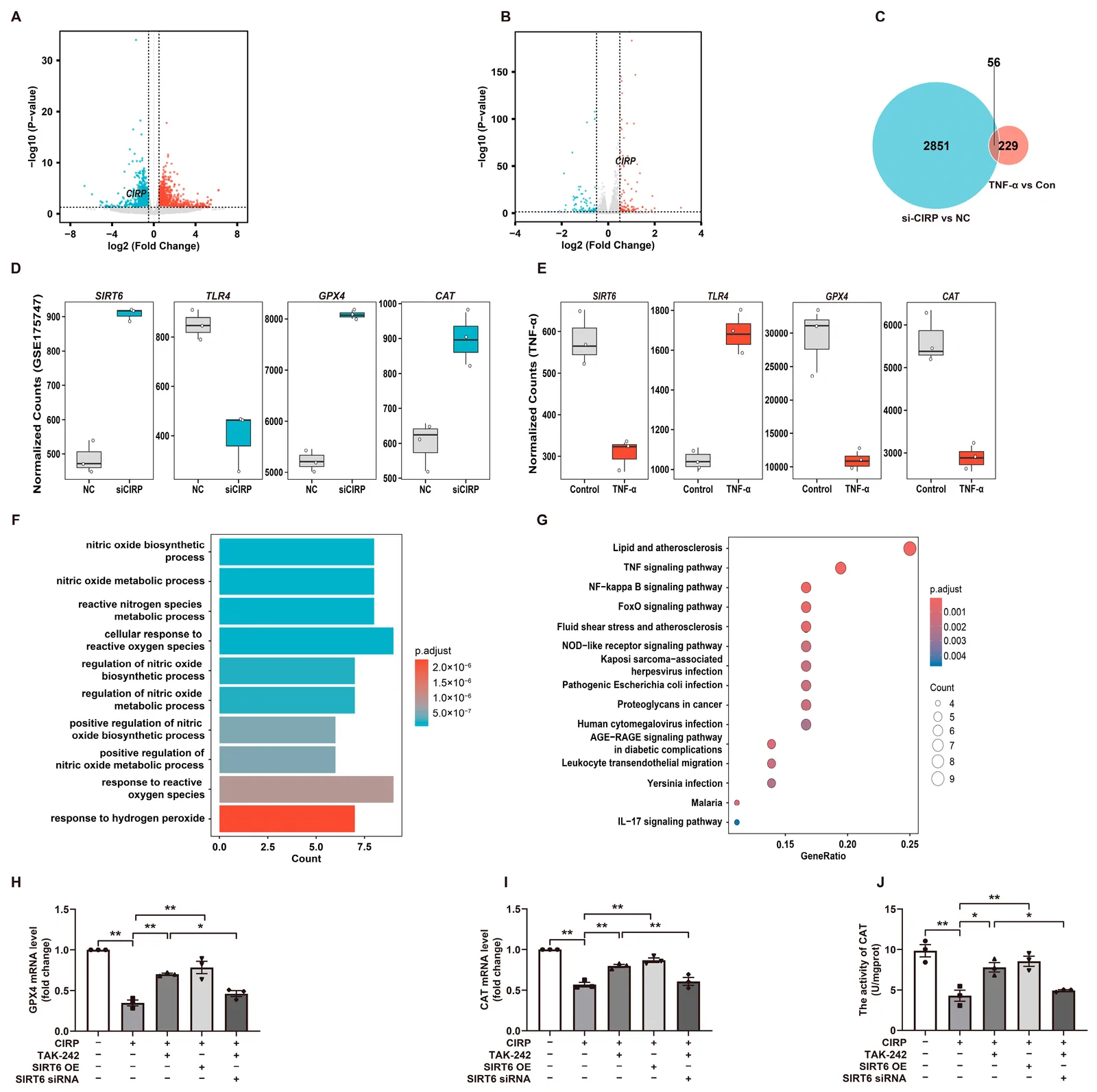
Exploratory transcriptomic analysis and experimental validation of CIRP-associated inflammatory and oxidative stress signatures. (**A**) Volcano plot of the CIRP-knockdown RNA-seq dataset. (**B**) Volcano plot of the TNF-α-stimulated HUVEC RNA-seq dataset. Red dots indicate significantly upregulated genes, blue dots indicate significantly downregulated genes, and gray dots indicate genes without significant differential expression. (**C**) Venn diagram showing 56 overlapping genes between the two datasets. The complete list of 56 overlapping genes is provided in Supplementary Table S5. (**D**,**E**) Expression patterns of SIRT6, TLR4, GPX4, and CAT in the CIRP-knockdown dataset and the TNF-α-stimulated HUVEC dataset. In the box plots, gray indicates the corresponding control group, blue indicates the si-CIRP group, and red indicates the TNF-α-treated group. (**F**) GO biological process enrichment analysis. (**G**) KEGG pathway enrichment analysis. (**H,I**) GPX4 and CAT mRNA expressions were measured by RT-qPCR in HUVECs treated with CIRP in the presence or absence of TAK-242, SIRT6 overexpression, or SIRT6 siRNA as indicated, *n* = 3. (**J**) CAT activity was measured using commercial assay kits, *n* = 3. Data are presented as mean ± SEM. Dots indicate independent biological replicates. Comparisons among multiple groups were performed by one-way ANOVA. * *p* < 0.05, ** *p* < 0.01.

To complement the experimental findings, we performed an exploratory comparative transcriptomic analysis using a publicly available CIRP-knockdown dataset and our in-house TNF-α-stimulated HUVEC dataset. Because these datasets were generated from different biological contexts, this analysis was intended to identify overlapping inflammatory and oxidative stress-related signatures rather than direct CIRP-regulated endothelial target genes. Differential expression analyses revealed distinct transcriptional changes in both the CIRP-knockdown dataset and the TNF-α-stimulated HUVEC dataset (Figure 7A,B). By intersecting the differentially expressed genes from the two datasets, we identified 56 overlapping genes (Figure 7C). The complete list of these overlapping genes is provided in Supplementary Table S5. Among these overlapping genes, SIRT6, TLR4, GPX4, and CAT showed expression patterns broadly consistent with our experimental observations. In the CIRP-knockdown dataset, SIRT6, GPX4, and CAT were increased, whereas TLR4 was decreased (Figure 7D). In contrast, in the TNF-α-stimulated HUVEC dataset, SIRT6, GPX4, and CAT were decreased, whereas TLR4 was increased (Figure 7E). Functional enrichment analysis suggested that the overlapping genes were related to redox and inflammatory processes. GO biological process analysis showed enrichment in nitric oxide biosynthetic and metabolic processes, reactive oxygen species metabolic processes, cellular responses to oxidative stress, and responses to hydrogen peroxide (Figure 7F). KEGG pathway analysis showed enrichment in lipid and atherosclerosis, TNF signaling, NF-κB signaling, NOD-like receptor signaling (Figure 7G). To provide experimental support for the oxidative stress-related transcriptomic signatures, we examined GPX4 and CAT expression in HUVECs. CIRP stimulation reduced GPX4 and CAT mRNA expression, whereas TAK-242 treatment and SIRT6 overexpression partially restored their expression (Figure 7H,I). SIRT6 silencing weakened the restorative effect of TAK-242. Consistently, CAT activity decreased after CIRP stimulation and was partially restored by TAK-242 or SIRT6 overexpression, whereas SIRT6 silencing reduced the effect of TAK-242 (Figure 7J). Overall, these exploratory transcriptomic findings suggest that CIRP-associated gene changes overlap with inflammatory and oxidative stress-related endothelial injury signatures. The additional assessment of GPX4, CAT, and CAT activity is consistent with antioxidant-related alterations observed under CIRP stimulation. However, this cross-context analysis does not establish direct CIRP-regulated endothelial target genes.

### 3.8. Clinical Analysis Provides Exploratory Evidence Linking Circulating CIRP to Coronary Atherosclerotic Burden

**Figure 8:**
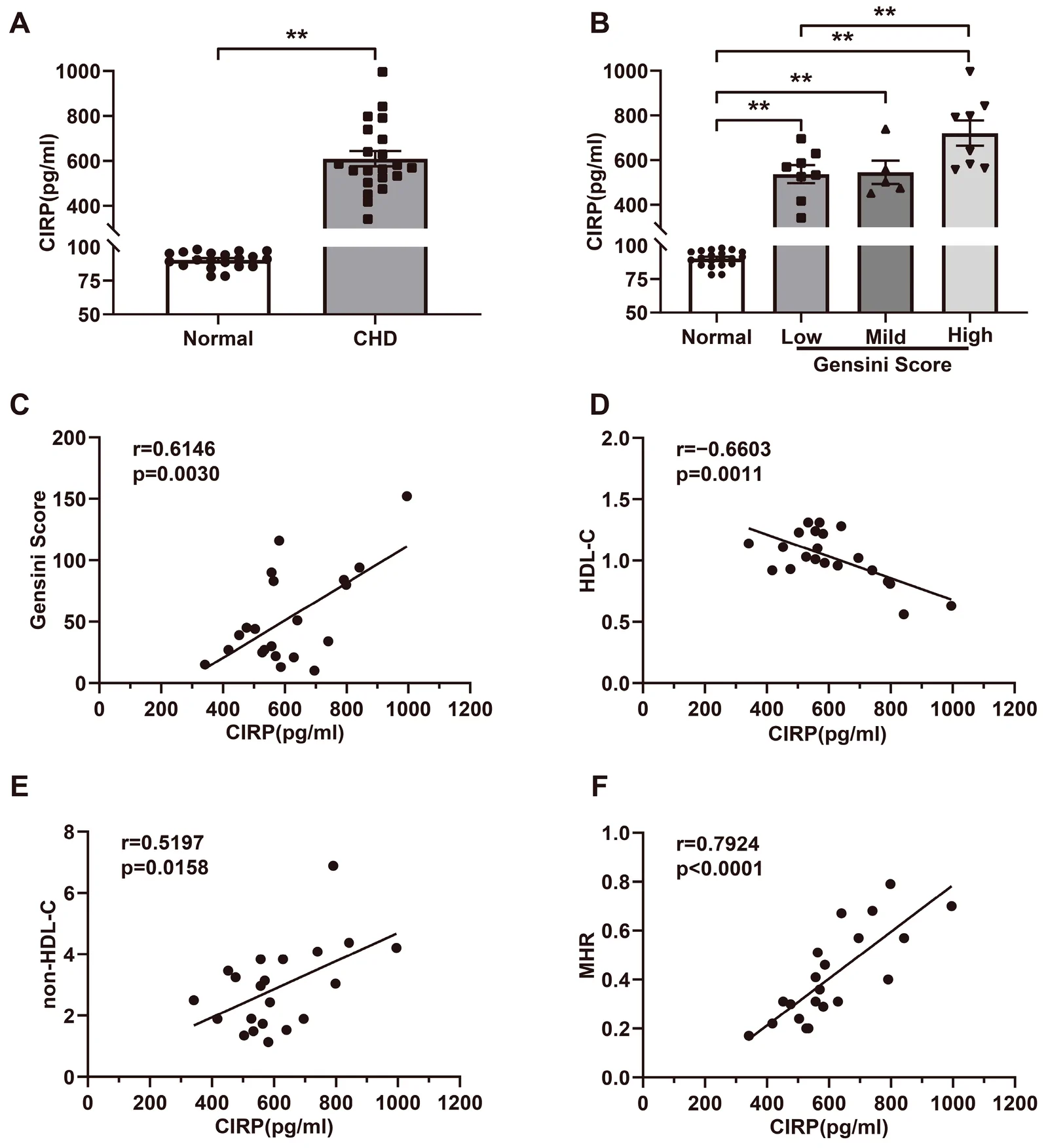
Exploratory clinical association between circulating CIRP and coronary atherosclerotic burden in patients with CHD. (**A**) Serum CIRP levels were measured using ELISA in the normal and CHD groups, Normal, *n* = 20 participants; CHD, *n* = 21 patients. (**B**) Serum CIRP levels across different CHD severity grades. (**C**–**F**) Correlations of serum CIRP levels with (**C**) Gensini score, (**D**) HDL-C, (**E**) non-HDL-C, and (**F**) monocyte-to-HDL-C ratio (MHR). Correlation analyses were performed in patients with CHD (*n* = 21). Data are presented as mean ± SEM. Dots indicate individual participants. Differences between two groups were analyzed by an unpaired Student’s *t*-test. Comparisons among multiple groups were performed by one-way ANOVA. Correlation analyses were conducted using Pearson’s correlation coefficient. ** *p* < 0.01.

Baseline clinical characteristics are summarized in Supplementary Tables S2 and S3. Compared with healthy examination controls, patients with CHD differed in age and sex distribution and in several lipid and inflammatory cell-related parameters. The CHD group had higher levels of TC, LDL-C, atherogenic index, neutrophil count, monocyte count, MHR, and NHR, together with lower HDL-C, lymphocyte count, and LHR. Serum CIRP levels were higher in patients with CHD than in healthy examination controls (Figure 8A). When patients with CHD were stratified according to Gensini score, CIRP levels were elevated across the CHD subgroups compared with controls and showed a tendency to increase with greater coronary atherosclerotic burden (Figure 8B). Correlation analyses were subsequently performed within the CHD group. Serum CIRP levels were positively correlated with Gensini score, suggesting a preliminary relationship between circulating CIRP and coronary lesion severity (Figure 8C). CIRP was also negatively correlated with HDL-C and positively correlated with non-HDL-C and MHR (Figure 8D–F). To further explore whether the association between CIRP and Gensini score was influenced by selected clinical variables, we performed exploratory multivariable linear regression analyses. Given the limited sample size, parsimonious models were used to reduce the risk of overfitting. The positive association between CIRP and Gensini score was retained after adjustment for age and after adjustment for age, HDL-C, and LDL-C. A similar association was observed in an exploratory model including age, systolic blood pressure, HDL-C, LDL-C, and neutrophil count. In the model adjusted for age and MHR, the association remained positive but was attenuated and showed borderline statistical significance (Supplementary Table S4). These results should therefore be interpreted cautiously and do not establish CIRP as an independent predictor of coronary atherosclerotic burden. To provide a broader overview of the clinical correlation pattern, CIRP and other clinical variables were further examined using correlation heatmaps (Figure S7A,B). CIRP showed correlations with several inflammatory cell-related variables, including monocyte, lymphocyte, and neutrophil counts and their derived ratios. However, given the limited cohort size and baseline differences between the CHD and control groups, these clinical findings should be regarded as exploratory. Collectively, the observations suggest a possible relationship between circulating CIRP, coronary atherosclerotic burden, and inflammatory cell-related indices in patients with CHD, which requires validation in larger cohorts.

## 4. Discussion

In the present study, we examined the relationship between CIRP and redox-inflammatory endothelial injury under atherosclerosis-related conditions. Extracellular CIRP has been recognized as a stress-responsive DAMP in acute inflammatory injury [2,21]. However, its relevance to chronic atherosclerosis-related vascular injury remains incompletely defined. In our HFD-fed ApoE^−/−^ mice, circulating CIRP levels were elevated and positively associated with atherosclerotic plaque burden. A preliminary time-course analysis also showed higher serum CIRP levels at later stages of HFD feeding. Nevertheless, plaque burden was not evaluated at the intermediate time points. These data therefore do not establish that circulating CIRP increased in parallel with lesion progression. At 12 weeks, elevated circulating CIRP occurred concurrently with systemic redox imbalance, impaired NO/eNOS-related endothelial function, and vascular inflammation. Because the 12-week HFD group was compared with mice at baseline (0 weeks) rather than with age-matched chow-fed controls, the effects of aging, HFD exposure, and lesion development could not be separated. In addition, no in vivo loss-of-function or gain-of-function experiments targeting CIRP were performed. Therefore, the animal findings should be interpreted as associative. Interestingly, aortic CIRP protein and mRNA levels were reduced despite the increase in circulating CIRP. This divergence suggests that circulating and aortic tissue CIRP may be regulated differently during chronic atherosclerosis-related stress, possibly through altered CIRP release, changes in local transcription, or differences in protein turnover. These possibilities warrant further investigation. In HUVECs, CIRP exposure was accompanied by redox imbalance, inflammatory responses, cellular injury, and impaired NO-/eNOS-related function. The concentration range was informed by previous studies using recombinant CIRP in endothelial models [7,9,10] and by our preliminary dose–response screening. Among the concentrations tested, 1000 ng/mL was the lowest concentration that produced a statistically significant reduction in cell viability. However, this concentration was substantially higher than the circulating CIRP levels measured in the animal and clinical samples. Cellular experiments should therefore be interpreted as a high-exposure experimental model rather than as a physiological exposure model. Overall, these findings support an association between extracellular CIRP and redox-inflammatory endothelial injury in HFD-fed ApoE^−/−^ mice and HUVECs. They also raise the question of how oxidative stress, inflammatory responses, and impaired NO/eNOS function are interconnected in this setting.

Oxidative stress is closely involved in endothelial injury and atherosclerotic lesion development. In ApoE^−/−^ mice, genetic disruption of NADPH oxidase components reduces vascular ROS production and attenuates atherosclerotic lesion formation, whereas Nox2 deficiency also reduced vascular superoxide production, preserved NO bioavailability, and limited early plaque formation [22,23]. In our HFD-fed ApoE^−/−^ mice, serum MDA levels were increased, while GSH-Px and CAT activities were reduced. This pattern is consistent with increased lipid peroxidation and impaired antioxidant capacity. Previous endothelial studies also provide a potential link between extracellular CIRP and redox injury. In primary mouse lung vascular endothelial cells, recombinant CIRP activated NADPH oxidase and promoted inflammatory endothelial injury [8]. Consistent with this observation, CIRP exposure increased intracellular ROS and MDA levels and reduced GSH-Px activity in our HUVECs. GPX4 and CAT expression and CAT activity were also decreased. These results support an association between CIRP exposure and endothelial redox imbalance. However, the cellular source of ROS and the specific antioxidant pathways involved were not directly determined in the present study. Redox imbalance is closely related to reduce NO bioavailability. Therefore, we next considered the NO/eNOS axis. Previous studies showed that eNOS deficiency accelerated lesion development in Western diet-fed ApoE^−/−^ mice [24]. More recent genetic evidence using gain-of-function eNOS mutants also showed that eNOS activation was associated with lower atherosclerotic indices [25]. In our HFD-fed ApoE^−/−^ mice, serum NO levels, eNOS activity, and aortic eNOS expression were reduced. These findings are consistent with impairment of NO-/eNOS-related endothelial function in the atherosclerotic animals. Direct evidence linking extracellular CIRP to endothelial eNOS regulation remains limited. In the present study, CIRP exposure reduced NO production, eNOS activity, and eNOS protein expression in HUVECs in concentration- and time-dependent manners. Thus, our findings extend the current CIRP literature to the endothelial NO/eNOS axis. However, the molecular basis of these changes remains to be established. Moreover, vascular reactivity and ex vivo vasorelaxation were not assessed. The NO/eNOS findings should therefore be interpreted as biochemical and cellular evidence rather than as direct evidence of impaired vascular responsiveness.

Inflammation represents another interconnected component of endothelial dysfunction. In this context, the distinct roles of eNOS and iNOS should be considered. Whereas eNOS-derived NO generally supports endothelial homeostasis, iNOS can participate in inflammatory endothelial injury. In TNF-α-treated HUVECs, iNOS deficiency attenuated ROS accumulation, NF-κB nuclear translocation, IL-6 expression, and adhesion molecule expression [26]. In our present study, aortic TNF-α, IL-1β and IL-6 expression was increased in HFD-fed ApoE^−/−^ mice. In HUVECs, CIRP exposure increased IL-1β secretion and elevated TNF-α and IL-1β mRNA expression. Previous work has linked CIRP-related inflammatory responses to NF-κB signaling. For example, the CIRP-derived inhibitory peptide C23 reduced TNF-α release and NF-κB nuclear translocation in CIRP-stimulated macrophages [27]. In our HUVEC model, CIRP exposure increased NF-κB p65 phosphorylation and enhanced THP-1 adhesion. These findings extend previous macrophage observations in CIRP-exposed human endothelial cells. However, NF-κB inhibition or genetic silencing was not performed. Therefore, the present data do not establish that NF-κB activation mediated downstream inflammatory responses. Taken together, the cellular findings support a coordinated phenotype involving redox imbalance, inflammatory responses, and impaired NO/eNOS-related function following CIRP exposure. The temporal and causal relationships among these processes remain to be defined.

TLR4 signaling has been implicated in the development of atherosclerosis. Genetic deficiency of TLR4 or its downstream adaptor MyD88 reduced aortic plaque burden, lipid deposition, and macrophage accumulation in ApoE^−/−^ mice [28]. TLR4 was also reported to contribute to early intimal foam-cell accumulation at lesion-prone aortic sites [29]. In our HFD-fed ApoE^−/−^ mice, TLR4 protein and mRNA expression were increased in aortic tissues. Immunofluorescence staining further showed an increased TLR4 signal in CD31-positive regions within atherosclerotic lesions. These findings are consistent with possible endothelial involvement of TLR4-related signaling under atherosclerosis-related conditions. However, increased receptor expression alone does not demonstrate functional TLR4 activation. Extracellular CIRP was initially identified as a DAMP whose inflammatory activity involved the TLR4/MD2 complex [2]. Previous work showed that CIRP reduced GPX4 expression and increased lipid ROS in macrophages. These effects were attenuated by TLR4 neutralization or TLR4 deficiency [30]. In our HUVEC model, pharmacological TLR4 inhibition with TAK-242 reduced CIRP-associated ROS accumulation and MDA production and partially restored GSH-Px activity. These findings extend previous CIRP–TLR4 redox observations to human endothelial cells. Nevertheless, the upstream source of ROS was not determined, and the TAK-242 results alone do not establish that these redox changes were specifically mediated by TLR4. TLR4 signaling has also been linked to impaired endothelial NO/eNOS homeostasis in non-CIRP vascular models. Under oscillatory shear stress, TLR4-related signaling promoted NOX2-dependent oxidative stress and eNOS suppression, whereas TLR4 inhibition restored NO levels [31]. In our study, TAK-242 partially restored NO production, eNOS activity, and eNOS protein expression in CIRP-exposed HUVECs. Direct evidence linking CIRP, TLR4, and endothelial eNOS regulation remains limited. Therefore, our findings provide pharmacological evidence for a possible relationship between TLR4-related signaling and impaired NO/eNOS function following CIRP exposure. However, they do not establish that TLR4 directly regulates eNOS in this experimental system. The inflammatory effects of CIRP have been more extensively linked to TLR4. For example, CIRP increased IL-6 and TNF-α production in wild-type Kupffer cells, whereas these responses were reduced in TLR4-deficient cells [32]. In human endothelial models, TAK-242 and TLR4 silencing have also produced concordant reductions in NF-κB-related activation, adhesion molecule expression, and monocyte adhesion, although these studies used stimuli other than CIRP [33]. In our HUVECs, TAK-242 attenuated CIRP-associated LDH release, IL-1β secretion, and TNF-α and IL-1β mRNA expression. These findings support the possible involvement of TLR4-related signaling in the inflammatory and cytotoxic responses observed after CIRP exposure. However, they do not establish complete receptor specificity or prove that all downstream inflammatory changes were mediated through TLR4.

The TAK-242 concentration used in this study was selected on the basis of previous mechanistic and endothelial studies. TAK-242 has been shown to bind selectively to TLR4 and interfere with its interactions with the adaptor proteins TIRAP and TRAM [34]. Previous endothelial studies also showed concordant effects of TAK-242 and TLR4 silencing [33]. In our experiments, 1 μM TAK-242 did not reduce HUVEC viability when administered alone and partially restored viability during CIRP exposure. Thus, the observed protection was unlikely to result from overt TAK-242 cytotoxicity. Nevertheless, the absence of cytotoxicity does not exclude off-target or TLR4-independent effects. Pharmacological inhibition therefore cannot replace genetic validation. Future studies using endothelial TLR4 knockdown, endothelial-specific TLR4 deletion, or TLR4-deficient atherosclerosis-prone mice will be required to establish receptor specificity and in vivo relevance. Finally, TLR4 may not be the only receptor involved in extracellular CIRP responses. The original receptor-screening study showed that recombinant CIRP bound TLR2 and AGER/RAGE in addition to the TLR4/MD2 complex. However, macrophage TNF-α responses were abolished in TLR4-deficient cells but preserved in TLR2- or RAGE-deficient cells, indicating predominant functional dependence on TLR4 in that experimental context [2]. Subsequent studies identified TREM-1 and IL-6R as functional CIRP receptors in myeloid and other cell-specific contexts [35,36]. Although we additionally assessed TLR2, TREM-1, IL-6R, and AGER expression, expression profiling alone cannot establish receptor activation or functional involvement. Therefore, contributions from these receptors cannot be excluded in CIRP-exposed endothelial cells and require receptor-specific functional studies.

SIRT6 has been implicated as a protective regulator of endothelial function and atherosclerosis. SIRT6 overexpression was reported to reduce atherosclerotic lesion formation and inflammatory responses in HFD-fed ApoE^−/−^ mice and to suppress pyroptotic injury in ox-LDL-treated HUVECs [18]. Our previous study further showed that endothelial-specific SIRT6 deficiency aggravated atherosclerotic lesion development and impaired endothelial barrier function in HFD-fed ApoE^−/−^ mice [15]. In the present study, SIRT6 protein and mRNA levels were reduced in aortic tissues from HFD-fed ApoE^−/−^ mice and in CIRP-exposed HUVECs. The cellular findings extend previous observations by linking CIRP exposure to reduced endothelial SIRT6 expression. However, the animal findings remain associative and do not establish that circulating CIRP caused the reduction in aortic SIRT6. With respect to redox homeostasis, multi-omics evidence has suggested that SIRT6 alleviates endothelial oxidative stress by maintaining endoplasmic reticulum protein homeostasis [37]. In our HUVEC model, SIRT6 overexpression partially reduced CIRP-associated ROS accumulation and MDA production and partially restored GSH-Px and CAT activities. It also attenuated the reductions in GPX4 and CAT expression. These findings are consistent with an antioxidant role for SIRT6 in CIRP-exposed endothelial cells. Nevertheless, the rescue was incomplete, and the molecular pathways connecting SIRT6 to these antioxidant systems were not directly examined. Consistent with these observations and with our previous findings in cholesterol crystal-exposed endothelial cells [16], SIRT6 overexpression partially improved NO production, eNOS activity, and eNOS protein expression in CIRP-exposed HUVECs. It also reduced LDH release, IL-1β secretion, TNF-α and IL-1β mRNA expression, NF-κB p65 phosphorylation, and THP-1 adhesion. However, these responses were improved rather than fully normalized. Moreover, the present data do not establish that SIRT6 directly regulates eNOS or NF-κB in this experimental system. The complementary SIRT6 silencing results were endpoint-dependent. Under basal conditions, SIRT6 knockdown increased LDH release and reduced NO production and eNOS activity, supporting an endogenous protective role for SIRT6. Under CIRP exposure, additional SIRT6 knockdown further increased LDH release and IL-1β secretion. However, it did not further reduce NO production, eNOS activity, or eNOS expression and did not further increase TNF-α or IL-1β mRNA levels. The absence of an additional change in these endpoints may reflect floor or ceiling effects, limited dynamic range, or pathway saturation, because CIRP exposure alone had already substantially altered these measurements. Therefore, SIRT6 depletion selectively aggravated some, but not all, components of CIRP-associated endothelial injury.

We next examined the relationship between pharmacological TLR4 inhibition and SIRT6 regulation. CIRP exposure was associated with a faster decline in SIRT6 protein during cycloheximide treatment. TAK-242 partially restored SIRT6 mRNA and protein abundance and slowed the loss of SIRT6 protein during the cycloheximide chase. These findings are consistent with TLR4-related signaling contributing to CIRP-associated SIRT6 downregulation at both the expression and protein stability levels. However, the changes in SIRT6 mRNA do not establish direct transcriptional regulation. Promoter activity, transcription-factor binding, and mRNA stability were not examined. Conversely, SIRT6 overexpression did not significantly alter TLR4 mRNA or protein expression under the conditions tested. This finding argues against a simple feedback effect of SIRT6 on TLR4 abundance. However, it does not exclude potential effects of SIRT6 on TLR4 activation, adaptor recruitment, or downstream signaling. Thus, the reciprocal expression results are compatible with a directional relationship from TLR4-related signaling toward SIRT6, but they do not establish a direct or linear regulatory pathway. In the combination experiments, SIRT6 silencing partially weakened the TAK-242-associated improvements in NO/eNOS-related and redox indices. It also partially offset the TAK-242-associated reductions in LDH release, inflammatory responses, NF-κB p65 phosphorylation, and THP-1 adhesion. This partial reversal supports a functional contribution of SIRT6 to the protection associated with pharmacological TLR4 inhibition. However, SIRT6 should not be regarded as the sole downstream mediator of TLR4-related signaling. The residual protection observed after SIRT6 silencing suggests that additional SIRT6-independent inflammatory and redox-related mechanisms are also involved.

Acetylation is an important but context-dependent post-translational modification of eNOS. In ApoE^−/−^ mice, G004 treatment increased SIRT1 and eNOS expression and was accompanied by enhanced eNOS deacetylation and improved endothelial function [38]. In contrast, aspirin-induced acetylation of eNOS at Lys609 was reported to enhance calmodulin binding, eNOS activity, and NO production in HEK-293 cells and HUVECs [39]. These findings indicate that the functional consequences of eNOS acetylation depend on the modified lysine residue and experimental context. In the present study, CIRP exposure increased overall eNOS acetylation in HUVECs. This change occurred concurrently with reduced eNOS activity and NO production. Our findings therefore extend the current CIRP literature by identifying overall eNOS acetylation as a post-translational change associated with impaired NO-/eNOS-related function. However, the concurrent changes do not establish that increased acetylation directly caused the reduction in eNOS activity. Moreover, because only overall eNOS acetylation was measured, the functional direction of this modification cannot be inferred without identifying the specific lysine residues involved. We performed a preliminary in silico analysis that identified several putative acetylation sites, including Lys5, Lys607, and Lys731. However, these candidates were not experimentally validated and should not be interpreted as CIRP-, TLR4-, or SIRT6-regulated sites. Previous evidence provides a potential basis for considering SIRT6 in the regulation of eNOS acetylation. SIRT6 has been reported to associate with eNOS and to deacetylate Lys494, Lys497, and Lys504, thereby enhancing eNOS activity and NO production [20]. However, direct evidence linking extracellular CIRP or TLR4-related signaling to eNOS acetylation remains limited. In our HUVEC model, pharmacological TLR4 inhibition with TAK-242 attenuated the CIRP-associated increase in overall eNOS acetylation. Reciprocal co-immunoprecipitation also detected SIRT6 and eNOS within the same protein complex. In addition, SIRT6 silencing did not further increase overall eNOS acetylation during CIRP exposure, but it partially weakened the ability of TAK-242 to reduce this modification. This pattern suggests that SIRT6 may contribute to the changes in eNOS acetylation associated with pharmacological TLR4 inhibition, rather than acting as the sole determinant of eNOS acetylation. The absence of a further increase after SIRT6 silencing may reflect a ceiling effect, limited assay dynamic range, or the contribution of other acetylation-regulating enzymes. Furthermore, site-specific acetylation mapping, eNOS acetylation-deficient or acetylation-mimetic mutants, catalytically inactive SIRT6 constructs, and purified-protein deacetylation assays were not performed. Therefore, the present findings support an association among pharmacological TLR4 inhibition, SIRT6 status, and overall eNOS acetylation, but they do not establish a direct linear TLR4-SIRT6-eNOS deacetylation mechanism.

Beyond the targeted TLR4/SIRT6 and eNOS-related experiments, we performed an exploratory transcriptomic comparison to examine whether broader redox- and inflammation-related signatures were shared across different experimental contexts. This analysis compared a public CIRP-silencing dataset with our TNF-α-treated HUVEC dataset and identified 56 overlapping genes. Functional enrichment of these genes highlighted pathways related to oxidative stress, ROS metabolism, nitric oxide-related processes, TNF signaling, NF-κB signaling, and NOD-like receptor signaling. However, this analysis was cross-context and hypothesis-generating. The two datasets differed in disease context and perturbation strategy. In addition, the overlap was based on shared gene identity rather than concordant direction of expression. Therefore, the overlapping genes should not be interpreted as direct endothelial targets of CIRP or as evidence of a unified CIRP-regulated transcriptional program. The observed changes in NF-κB p65 phosphorylation, GPX4 and CAT expression, and CAT activity provided targeted experimental support for selected inflammatory and antioxidant signatures highlighted by the analysis. However, these targeted measurements do not validate the entire transcriptomic network. Direct transcriptomic profiling of CIRP-exposed endothelial cells will be required to define CIRP-responsive endothelial pathways more precisely.

Clinical evidence regarding circulating CIRP in coronary atherosclerotic disease remains limited. In a previous cohort of patients with acute coronary syndrome, higher plasma CIRP levels were associated with adverse cardiovascular outcomes [4]. However, that study focused on prognosis after an acute coronary event rather than on angiographic atherosclerotic burden. In our cohort, serum CIRP levels were higher in patients with CHD than in control participants. CIRP levels also differed across Gensini score-defined subgroups and were positively correlated with Gensini score within the CHD group. These findings provide preliminary evidence for a possible relationship between circulating CIRP and angiographic coronary disease burden. However, the between-group comparison was unadjusted, and the CHD and control groups differed in age and sex distribution. The observed group difference should therefore be interpreted cautiously. In our CHD cohort, circulating CIRP was also inversely correlated with HDL-C and positively correlated with non-HDL-C and MHR. Additional correlation analyses also showed positive associations between CIRP and several inflammatory cell-derived indices. Nevertheless, these correlations do not establish whether CIRP contributes to dyslipidemia or inflammation, is released in response to vascular injury, or reflects a broader metabolic-inflammatory state. We further performed exploratory multivariable analyses within the CHD group. The positive association between circulating CIRP and Gensini score remained detectable after adjustment for selected covariates in several parsimonious models. However, the association was attenuated when MHR was included in the model. This attenuation suggests that the CIRP–Gensini relationship may partly overlap with inflammatory status. It should not be interpreted as evidence that MHR mediates this relationship, because formal mediation analysis was not performed. Moreover, the small number of patients limited the number of covariates that could be included without substantial risk of model overfitting. Accordingly, these analyses do not establish circulating CIRP as an independent predictor of coronary atherosclerotic severity. The clinical findings should be regarded as preliminary and supportive rather than causal. The analysis was limited by the small sample size, retrospective design, demographic imbalance, and residual confounding. The within-CHD multivariable models did not address the age and sex differences between the CHD and control groups. Larger prospective studies with matched controls are needed to determine whether circulating CIRP provides information beyond established inflammatory and cardiovascular risk factors.

Several limitations should be acknowledged. First, the animal study did not include in vivo CIRP loss-of-function or gain-of-function experiments in atherosclerosis-prone models. Therefore, the animal findings should be interpreted as associative. In addition, the CIRP concentration used in HUVECs exceeded the circulating levels measured in vivo; thus, the cellular experiments should be regarded as a high-exposure mechanistic model. Second, TLR4 involvement was assessed primarily using TAK-242. Although TAK-242 is a well-established pharmacological inhibitor, genetic TLR4 silencing or deletion will be required to confirm receptor specificity and exclude potential off-target effects. Moreover, TLR4 should not be considered the only receptor involved in extracellular CIRP responses. Third, only overall eNOS acetylation was examined. Future studies using site-specific mapping, functional eNOS mutants, and direct deacetylation assays are needed to clarify the contribution of SIRT6-related eNOS regulation. Fourth, the transcriptomic analysis was exploratory and based on datasets generated under different experimental conditions. The overlapping genes should therefore be interpreted as hypothesis-generating rather than as direct endothelial targets of CIRP, and direct transcriptomic profiling of CIRP-exposed endothelial cells is needed. Finally, the clinical analysis was limited by the small retrospective cohort, demographic imbalance, and residual confounding. Larger prospective studies with appropriately matched controls will be required to validate these observations. Despite these limitations, the present study integrates in vivo, in vitro, bioinformatic, and preliminary clinical evidence and may provide a framework for further investigation of CIRP-related redox-inflammatory endothelial injury and TLR4-/SIRT6-associated signaling in atherosclerosis.

## 5. Conclusions

In conclusion, this study provides preliminary evidence linking extracellular CIRP to redox-inflammatory endothelial injury under atherosclerosis-related conditions. In HFD-fed ApoE^−/−^ mice, circulating CIRP was elevated and associated with plaque burden. CIRP exposure in HUVECs was accompanied by oxidative imbalance, inflammatory responses and impaired NO-/eNOS-related function. Pharmacological TLR4 inhibition and SIRT6 gain- and loss-of-function experiments suggest that TLR4-related signaling and SIRT6 may participate in these endothelial changes. The observed increase in overall eNOS acetylation may represent an associated post-translational event rather than a defined causal mechanism. Exploratory transcriptomic findings and preliminary clinical associations provide additional context for experimental observations. Taken together, these findings support CIRP as a potential contributor to redox-inflammatory endothelial injury and suggest that TLR4-/SIRT6-associated signaling may participate in this process.

**Table 2:** Gene accession numbers of genes mentioned.

Gene/Protein Mentioned	Official Gene Symbol	Full Name	NCBI Gene ID (Homo Sapiens)	NCBI Gene ID (Mus Musculus)
CIRP	CIRBP/Cirbp	cold inducible RNA binding protein	1153	12696
TLR4	TLR4/Tlr4	toll like receptor 4	7099	21898
SIRT6	SIRT6/Sirt6	sirtuin 6	51548	50721
TLR2	TLR2/Tlr2	toll like receptor 2	7097	24088
eNOS/NOS3	NOS3/Nos3	nitric oxide synthase 3	4846	18127
GPX4	GPX4/Gpx4	glutathione peroxidase 4	2879	625249
NLRP3	NLRP3/Nlrp3	NLR family pyrin domain containing 3	114548	216799
TNF-α	TNF/Tnf	tumor necrosis factor	7124	21926
iNOS	NOS2/Nos2	nitric oxide synthase 2	4843	18126
MMP-2	MMP2/Mmp2	matrix metallopeptidase 2	4313	17390
IL-1β	IL1B/Il1b	interleukin 1 beta	3553	16176
IL-6	IL6/Il6	interleukin 6	3569	16193
CD31	PECAM1/Pecam1	platelet and endothelial cell adhesion molecule 1	5175	18613
CAT	CAT/Cat	catalase	847	12359
HMGB1	HMGB1/Hmgb1	high mobility group box 1	3146	15289
HSP70	HSPA1A/Hspa1a	heat shock protein family A (Hsp70) member 1A	3303	15511
SOD2	SOD2/Sod2	superoxide dismutase 2	6648	20656
GPX-1	GPX1/Gpx1	glutathione peroxidase 1	2876	14775
TREM-1	TREM1/Trem1	triggering receptor expressed on myeloid cells 1	54210	58217
AGER/RAGE	AGER/Ager	advanced glycosylation end-product specific receptor	177	11596

## Figures and Tables

**Table 1:** Primer sequences used for quantitative real-time PCR.

Gene	Primer	Sequence (5′–3′)
Mouse *GAPDH*	R	TTGCTGTTGAAGTCGCAGGAG
	F	TGTGTCCGTCGTGGATCTGA
Mouse *CIRP*	F	CGAAGTGGTGGTGGTAAAGGACAG
	R	TGATCTGCCGCCCGTCCAC
Mouse *TLR4*	F	CCGCTTTCACCTCTGCCTTCAC
	R	ACCACAATAACCTTCCGGCTCTTG
Mouse *SIRT6*	F	CATGGGCTTCCTCAGCTTCC
	R	CACTTGGGACATTCCTCTACAAACA
Mouse *TNF-α*	F	CAGGCGGTGCCTATGTCTC
	R	CGATCACCCCGAAGTTCAGTAG
Mouse *IL-1β*	F	CTGTGTCTTTCCCGTGGACC
	R	CAGCTCATATGGGTCCGACA
Mouse *IL-6*	R	AATCAGAATTGCCATTGCACAAC
	F	AGAGGAGACTTCACAGAGGATACC
Human *GAPDH*	R	GGCTGTTGTCATACTTCTCATGG
	F	GGAGCGAGATCCCTCCAAAAT
Human *TLR4*	F	TGAGGACTGGGTGAGAAATGAGC
	R	CTGCCATGTTTGAGCAATCTCAT
Human *SIRT6*	R	CTCTGCCAGTTTGTCCCTG
	F	CCCACGGAGTCTGGACCAT
Human *IL-1β*	R	CAGCTCATATGGGTCCGACA
	F	CTGTGTCTTTCCCGTGGACC
Human *TNF-α*	F	TCTTCTCGAACCCCGAGTGA
	R	ATGAGGTACAGGCCCTCTGA
Human *GPX4*	F	CAGTGAGGCAAGACCGAAGT
	R	CCGAACTGGTTACACGGGAA
Human *CAT*	F	TGAAGATGCGGCGAGACTTT
	R	GAGGGGTACTTTCCTGTGGC
Human *TLR2*	F	CTGTGCTCTGTTCCTGCTGA
	R	GATGTTCCTGCTGGGAGCTT
Human *TREM1*	F	AGGAGCCTCACATGCTGTTC
	R	AGTGGAGACATCGGCAGTTG
Human *IL6R*	F	CCCAGAAGTTCTCCTGCCAG
	R	CTGAACTTGCTCCCGACACT
Human *AGER*	F	TGGATGAAGGATGGTGTGCC
	R	CACAGCTGTAGGTTCCCTGG

## Data Availability

The publicly available CIRP-knockdown RNA-sequencing dataset analyzed in this study is deposited in the GEO under accession number: GSE175747, available accessed at https://www.ncbi.nlm.nih.gov/geo/query/acc.cgi?acc=GSE175747 (accessed on 16 December 2025). The corresponding raw sequencing data are available in the NCBI Sequence Read Archive (SRA) under accession number SRP321778 at https://www.ncbi.nlm.nih.gov/sra?term=SRP321778 (accessed on 16 December 2025). The TNF-α-stimulated HUVEC RNA-sequencing dataset was generated as part of an independent ongoing study and was used in the present study only for exploratory comparative analysis. This dataset is available from the corresponding author upon reasonable request. The remaining data supporting the findings of this study are contained within the article and its Supplementary Materials. De-identified clinical data may be available from the corresponding author upon reasonable request, subject to applicable ethical and institutional requirements.

## Supplementary Materials

The following supporting information can be downloaded at https://www.mdpi.com/article/10.3390/antiox15080943/s1, Figure S1: HFD feeding increases vascular inflammatory gene expression in ApoE^−/−^ mice. Figure S2: CIRP induces inflammation in HUVECs. Figure S3: SIRT6 overexpression suppresses CIRP-induced inflammation in endothelial cells. Figure S4: TLR4 inhibition suppresses CIRP-induced inflammation and restores SIRT6 expression in endothelial cells. Figure S5: Evaluation of alternative CIRP-associated receptors in HUVECs. Figure S6: Effects of SIRT6 knockdown on CIRP-induced endothelial injury and inflammation. Figure S7: Correlation heatmap analysis of clinical parameters in patients with CHD. Table S1: Gensini scoring system used to assess coronary atherosclerotic burden. Table S2: Baseline characteristics of the study participants. Table S3: Comparison of metabolic and inflammatory parameters among study groups. Table S4: Exploratory multivariable linear regression analyses for the association between circulating CIRP and Gensini score in patients with CHD. Table S5: List of 56 overlapping genes.

## Abbreviations

The following abbreviations are used in this manuscript:

CAT	Catalase
CC	Cholesterol crystals
CHD	Coronary heart disease
CIRP	Cold-inducible RNA-binding protein
COPD	Chronic obstructive pulmonary disease
DAMP	Damage-associated molecular pattern
eNOS	Endothelial nitric oxide synthase
GO	Gene ontology
GPX4	Glutathione peroxidase 4
GSH-Px	Glutathione peroxidase
H&E	Hematoxylin and eosin
HDL-C	High-density lipoprotein cholesterol
HFD	High-fat diet
HUVECs	Human umbilical vein endothelial cells
IL	Interleukin
iNOS	Inducible nitric oxide synthase
KEGG	Kyoto Encyclopedia of Genes and Genomes
LDH	Lactate dehydrogenase
LDL-C	Low-density lipoprotein cholesterol
MDA	Malondialdehyde
MMP-2	Matrix metalloproteinase-2
NADPH	Nicotinamide adenine dinucleotide phosphate
NLRP3	NOD-like receptor family pyrin domain-containing 3
NO	Nitric oxide
ox-LDL	Oxidized low-density lipoprotein
PRRs	pattern-recognition receptors
ROS	Reactive oxygen species
SIRT6	Sirtuin 6
SOD	Superoxide dismutase
TC	Total cholesterol
TLR2	Toll-like receptor 2
TLR4	Toll-like receptor 4
TREM-1	Triggering receptor expressed on myeloid cells-1
AGER/RAGE	Advanced glycosylation end-product specific receptor

## References

[1] Nishiyama H., Itoh K., Kaneko Y., Kishishita M., Yoshida O., Fujita J. (1997). A glycine-rich RNA-binding protein mediating cold-inducible suppression of mammalian cell growth. J. Cell Biol..

[2] Qiang X., Yang W.L., Wu R., Zhou M., Jacob A., Dong W., Kuncewitch M., Ji Y., Yang H., Wang H. (2013). Cold-inducible RNA-binding protein (CIRP) triggers inflammatory responses in hemorrhagic shock and sepsis. Nat. Med..

[3] Aziz M., Chaudry I.H., Wang P. (2025). Extracellular Cold-Inducible RNA-Binding Protein: Progress from Discovery to Present. Int. J. Mol. Sci..

[4] Ren X., Xie H., Zhang J., Jin X., Cui L., Chen L., Chen L., Zuo G. (2022). Prognostic Value of Plasma Cold-Inducible RNA-Binding Protein in Patients with Acute Coronary Syndrome. Dis. Markers.

[5] Wang L., Li R.F., Guan X.L., Liang S.S., Gong P. (2021). The Value of Extracellular Cold-Inducible RNA-Binding Protein (eCIRP) in Predicting the Severity and Prognosis of Patients After Cardiac Arrest: A Preliminary Observational Study. Shock.

[6] Li G., Yang L., Yuan H., Liu Y., He Y., Wu X., Jin X. (2016). Cold-inducible RNA-binding protein plays a central role in the pathogenesis of abdominal aortic aneurysm in a murine experimental model. Surgery.

[7] Tsai H.Y., Chien W.C., Chung C.H., Lin C.Y., Hsu Y.J., Tsai M.C., Hsu C.W., Tsai S.H. (2026). Cold exposure increases aortic dissection risk through extracellular cold inducible RNA binding protein and toll like receptor 4 signaling. Sci. Rep..

[8] Yang W.L., Sharma A., Wang Z., Li Z., Fan J., Wang P. (2016). Cold-inducible RNA-binding protein causes endothelial dysfunction via activation of Nlrp3 inflammasome. Sci. Rep..

[9] Takizawa S., Lee Y., Jacob A., Aziz M., Wang P. (2022). Neutrophil trogocytosis during their trans-endothelial migration: Role of extracellular CIRP. Mol. Med..

[10] Liu W., Wu D.H., Wang T., Wang M., Xu Y., Ren Y., Lyu Y., Wu R. (2025). CIRP contributes to multiple organ damage in acute pancreatitis by increasing endothelial permeability. Commun. Biol..

[11] Chen R.R., Li Y.Y., Wu J.W., Wang Y., Song W., Shao D., Gao W., Yu H. (2025). SIRT6 in health and diseases: From molecular mechanisms to therapeutic prospects. Pharmacol. Res..

[12] Wu K., Wang Y., Liu R., Wang H., Rui T. (2023). The role of mammalian Sirtuin 6 in cardiovascular diseases and diabetes mellitus. Front. Physiol..

[13] Greiten L.E., Zhang B., Roos C.M., Hagler M., Jahns F.P., Miller J.D. (2021). Sirtuin 6 Protects Against Oxidative Stress and Vascular Dysfunction in Mice. Front. Physiol..

[14] Liu R., Liu H., Ha Y., Tilton R.G., Zhang W. (2014). Oxidative stress induces endothelial cell senescence via downregulation of Sirt6. Biomed. Res. Int..

[15] Jin Z., Xiao Y., Yao F., Wang B., Zheng Z., Gao H., Lv X., Chen L., He Y., Wang W. (2020). SIRT6 inhibits cholesterol crystal-induced vascular endothelial dysfunction via Nrf2 activation. Exp. Cell Res..

[16] Zheng Z., Wang B., Lv X., Yao F., Gao H., Jin Z., Liu Y., Deng Y., Chen D., Ning X. (2021). Protective effect of SIRT6 on cholesterol crystal-induced endothelial dysfunction via regulating ACE2 expression. Exp. Cell Res..

[17] Yang L., Chen W., Li B., Hu Y., Lu H., Zhang P., Yang H., Zhang M., Pan D. (2023). Circular RNA circ_0026218 Suppressed Atherosclerosis Progression via miR-338-3p/SIRT6 Axis. Biomed. Res. Int..

[18] Huang J., Dong S., Wu Y., Yi H., Zhang W., Ai X. (2024). Sirtuin 6 Deacetylates Apoptosis-Associated Speck-Like Protein (ASC) to Inhibit Endothelial Cell Pyroptosis in Atherosclerosis. Int. Heart J..

[19] Gong T., Wang Q.D., Loughran P.A., Li Y.H., Scott M.J., Billiar T.R., Liu Y.T., Fan J. (2024). Mechanism of lactic acidemia-promoted pulmonary endothelial cells death in sepsis: Role for CIRP-ZBP1-PANoptosis pathway. Mil. Med. Res..

[20] Wang J., Liu Z., Lu J., Zou J., Ye W., Li H., Gao S., Liu P. (2023). SIRT6 regulates endothelium-dependent relaxation by modulating nitric oxide synthase 3 (NOS3). Biochem Pharmacol..

[21] Han J., Zhang Y., Ge P., Dakal T.C., Wen H., Tang S., Luo Y., Yang Q., Hua B., Zhang G. (2023). Exosome-derived CIRP: An amplifier of inflammatory diseases. Front. Immunol..

[22] Barry-Lane P.A., Patterson C., van der Merwe M., Hu Z., Holland S.M., Yeh E.T., Runge M.S. (2001). p47phox is required for atherosclerotic lesion progression in ApoE^−/−^ mice. J. Clin. Investig..

[23] Judkins C.P., Diep H., Broughton B.R., Mast A.E., Hooker E.U., Miller A.A., Selemidis S., Dusting G.J., Sobey C.G., Drummond G.R. (2010). Direct evidence of a role for Nox2 in superoxide production, reduced nitric oxide bioavailability, and early atherosclerotic plaque formation in ApoE^−/−^ mice. Am. J. Physiol. Heart Circ. Physiol..

[24] Kuhlencordt P.J., Gyurko R., Han F., Scherrer-Crosbie M., Aretz T.H., Hajjar R., Picard M.H., Huang P.L. (2001). Accelerated atherosclerosis, aortic aneurysm formation, and ischemic heart disease in apolipoprotein E/endothelial nitric oxide synthase double-knockout mice. Circulation.

[25] Nguyen T.D., Rahman N.T., Sessa W.C., Lee M.Y. (2023). Endothelial nitric oxide synthase (eNOS) S1176 phosphorylation status governs atherosclerotic lesion formation. Front. Cardiovasc. Med..

[26] Liu C., Lei S., Cai T., Cheng Y., Bai J., Fu W., Huang M. (2023). Inducible nitric oxide synthase activity mediates TNF-α-induced endothelial cell dysfunction. Am. J. Physiol. Cell Physiol..

[27] Zhang F., Brenner M., Yang W.L., Wang P. (2018). A cold-inducible RNA-binding protein (CIRP)-derived peptide attenuates inflammation and organ injury in septic mice. Sci. Rep..

[28] Michelsen K.S., Wong M.H., Shah P.K., Zhang W., Yano J., Doherty T.M., Akira S., Rajavashisth T.B., Arditi M. (2004). Lack of Toll-like receptor 4 or myeloid differentiation factor 88 reduces atherosclerosis and alters plaque phenotype in mice deficient in apolipoprotein E. Proc. Natl. Acad. Sci. USA.

[29] Higashimori M., Tatro J.B., Moore K.J., Mendelsohn M.E., Galper J.B., Beasley D. (2011). Role of toll-like receptor 4 in intimal foam cell accumulation in apolipoprotein E-deficient mice. Arterioscler. Thromb. Vasc. Biol..

[30] Shimizu J., Murao A., Nofi C., Wang P., Aziz M. (2022). Extracellular CIRP Promotes GPX4-Mediated Ferroptosis in Sepsis. Front. Immunol..

[31] Wang Z., Wang F., Kong X., Gao X., Gu Y., Zhang J. (2019). Oscillatory Shear Stress Induces Oxidative Stress via TLR4 Activation in Endothelial Cells. Mediat. Inflamm..

[32] Shimizu J., Murao A., Lee Y., Aziz M., Wang P. (2024). Extracellular CIRP promotes Kupffer cell inflammatory polarization in sepsis. Front. Immunol..

[33] Han Y., Liao X., Gao Z., Yang S., Chen C., Liu Y., Wang W.E., Wu G., Chen X., Jose P.A. (2016). Cardiac troponin I exacerbates myocardial ischaemia/reperfusion injury by inducing the adhesion of monocytes to vascular endothelial cells via a TLR4/NF-κB-dependent pathway. Clin. Sci..

[34] Matsunaga N., Tsuchimori N., Matsumoto T., Ii M. (2011). TAK-242 (resatorvid), a small-molecule inhibitor of Toll-like receptor (TLR) 4 signaling, binds selectively to TLR4 and interferes with interactions between TLR4 and its adaptor molecules. Mol. Pharmacol..

[35] Denning N.L., Aziz M., Murao A., Gurien S.D., Ochani M., Prince J.M., Wang P. (2020). Extracellular CIRP as an endogenous TREM-1 ligand to fuel inflammation in sepsis. JCI Insight.

[36] Zhou M., Aziz M., Denning N.L., Yen H.T., Ma G., Wang P. (2020). Extracellular CIRP induces macrophage endotoxin tolerance through IL-6R-mediated STAT3 activation. JCI Insight.

[37] Li P., Guo Z., Feng R., Wu N., Zhong X., Fang Z., Hu Y., Yu X., Zhao S., Zhao G. (2022). Multi-omics analysis reveals the regulation of SIRT6 on protein processing of endoplasmic reticulum to alleviate oxidative stress in endothelial cells. Clin. Transl. Med..

[38] Qian L., Ma L., Wu G., Yu Q., Lin H., Ying Q., Wen D., Gao C. (2017). G004, a synthetic sulfonylurea compound, exerts anti-atherosclerosis effects by targeting SIRT1 in ApoE^−/−^ mice. Vasc. Pharmacol..

[39] Jung S.B., Kim C.S., Naqvi A., Yamamori T., Mattagajasingh I., Hoffman T.A., Cole M.P., Kumar A., Dericco J.S., Jeon B.H. (2010). Histone deacetylase 3 antagonizes aspirin-stimulated endothelial nitric oxide production by reversing aspirin-induced lysine acetylation of endothelial nitric oxide synthase. Circ. Res..

