# Supplementary material for: CIRP Promotes Redox-Inflammatory Endothelial Injury in High-Fat Diet-Fed ApoE−/− Mice and HUVECs: Involvement of TLR4/SIRT6 Signaling
Source: Antioxidants (Basel). 2026 Jul 29;15(8):943. doi: 10.3390/antiox15080943 (PMC13509557; doi:10.3390/antiox15080943)
Supplement: Supplementary file 1 [file antioxidants-15-00943-s001.zip › figure legends.pdf]

Figure S1. HFD feeding increases vascular inflammatory gene expression in $ApoE^{-/-}$ mice.

(A-C) Relative mRNA levels of TNF-α (A), IL-1β (B) and IL-6 (C) in aortic tissues from $ApoE^{-/-}$ mice fed a high-fat diet (HFD) for 0 or 12 weeks, as determined by RT-qPCR, n=5. (D) Serum CIRP levels in $ApoE^{-/-}$ mice fed HFD for 0, 4, 8, or 12 weeks, as measured by ELISA, n=6. Data are presented as mean ± SEM. Dots indicate individual mice. Differences between two groups were analyzed by an unpaired Student's t-test. Comparisons among multiple groups were performed by one-way ANOVA. $*p < 0.05$, $**p < 0.01$.

Figure S2. CIRP induces inflammation in HUVECs.

(A-B) Level of IL-1β in culture supernatant was detected using commercially available kits. (C-D) Relative mRNA expression of TNF-α in HUVECs treated with different concentrations of CIRP (C) or stimulated with CIRP for the indicated time points (D), as determined by RT-qPCR. (E-F) IL-1β mRNA expression was measured after dose- and time-dependent CIRP treatment. Data are presented as mean ± SEM, n=3. Dots indicate independent biological replicates. Comparisons among multiple groups were performed by one-way ANOVA. $*p < 0.05$, $**p < 0.01$.

Figure S3. SIRT6 overexpression suppresses CIRP-induced inflammation in endothelial cells.

(A-B) SIRT6 overexpression efficiency after adenoviral transduction was confirmed by Western blotting and RT-qPCR. (C) IL-1β release in culture supernatant was detected by ELISA kits. (D-E) TNF-α and IL-1β mRNA expression was measured by RT-qPCR. (F-G) TLR4 mRNA and total protein expression in HUVECs treated with CIRP in the presence or absence of SIRT6 overexpression. Data are presented as mean ± SEM, n=3. Dots indicate independent biological replicates. Differences between two groups were analyzed by an unpaired Student's t-test. Comparisons among multiple groups were performed by one-way ANOVA. $*p < 0.05$, $**p < 0.01$.

Figure S4. TLR4 inhibition suppresses CIRP-induced inflammation and restores SIRT6 expression in endothelial cells.

(A) IL-1β secretion in the culture supernatant of HUVECs treated with CIRP in the presence or absence of the TLR4 inhibitor TAK-242, as measured by ELISA. (B-C) TNF-α and IL-1β mRNA expression was measured by RT-qPCR. (D-E) Effects of TAK-

242 on SIRT6 expression were detected by Western blotting and RT-qPCR. (F-G) SIRT6 protein stability was evaluated by cycloheximide (CHX) chase assay in HUVECs treated with vehicle, CIRP, or TAK-242 plus CIRP. Quantitative analysis of SIRT6 protein levels relative to 0 h is shown in (G). (H) HUVECs were treated with vehicle or increasing concentrations of TAK-242 for 25 h, and cell viability was assessed using the CCK-8 assay. (I) HUVECs were pretreated with vehicle or TAK-242 at the indicated concentrations for 1 h, followed by CIRP stimulation at 1000 ng/mL for 24 h. Cell viability was measured using the CCK-8 assay. Data are presented as mean ± SEM, *n*=3. Dots indicate independent biological replicates. Comparisons among multiple groups were performed by one-way ANOVA. The degradation curves were analyzed by two-way ANOVA. $*p < 0.05$, $**p < 0.01$.

Figure S5. Evaluation of alternative CIRP-associated receptors in HUVECs.

(A-B) TLR2 protein expression (H) and mRNA expression (I) in HUVECs treated with different concentrations of CIRP. (C-D) TLR2 protein expression (C) and mRNA expression (D) in HUVECs stimulated with CIRP for the indicated time points. (E-F) TREM-1 protein and mRNA levels in HUVECs treated with different concentrations of CIRP for 24 h. (G-H) TREM-1 protein and mRNA levels in HUVECs treated with CIRP for different durations. TREM-1 protein was weakly detectable in HUVECs, consistent with low endogenous expression. (I-J) IL6R mRNA levels in HUVECs treated with different concentrations of CIRP or for different durations. (K-L) AGER mRNA levels in HUVECs treated with different concentrations of CIRP or for different durations. Data are presented as mean ± SEM, *n*=3. Dots indicate independent biological replicates. Comparisons among multiple groups were performed by one-way ANOVA. $*P < 0.05$, $**P < 0.01$.

Figure S6. Effects of SIRT6 knockdown on CIRP-induced endothelial injury and inflammation.

(A-B) SIRT6 knockdown efficiency in HUVECs was determined by Western blotting and RT-qPCR, *n*=3. HUVECs were transfected with SIRT6 siRNA for 48 h and then were incubated with CIRP for 24 h. (C-E) LDH release, NO production, and eNOS activity were measured using assay kits, *n*=3. (F) eNOS protein expression in HUVECs was determined by Western blotting, *n*=5. (G) IL-1β secretion in culture supernatants was measured by ELISA, *n*=3. (H-I) TNF-α and IL-1β mRNA expression was analyzed by RT-qPCR, *n*=3. Data are presented as mean ± SEM. Dots indicate independent

biological replicates. Comparisons among multiple groups were performed by one-way ANOVA. *$p < 0.05$, **$p < 0.01$; ns, not significant.

Figure S7. Correlation heatmap analysis of clinical parameters in patients with CHD

(A) Unsupervised clustering heatmap showing Pearson correlation coefficients among other clinical parameters. Red indicates positive correlations, and blue indicates negative correlations. (B) Heatmap showing normalized levels of serum CIRP and other clinical variables across 21 patients with CHD. Patient IDs are shown on the right. LHR, lymphocyte-to-HDL-C ratio; NHR, neutrophil-to-HDL-C ratio; NLR, neutrophil-to-lymphocyte ratio; AI, atherogenic index; TC, total cholesterol; LDL-C, low-density lipoprotein cholesterol; sCr, serum creatinine; SBP, systolic blood pressure; DBP, diastolic blood pressure.
