# Supplementary material for: CIRP Promotes Redox-Inflammatory Endothelial Injury in High-Fat Diet-Fed ApoE−/− Mice and HUVECs: Involvement of TLR4/SIRT6 Signaling
Source: Antioxidants (Basel). 2026 Jul 29;15(8):943. doi: 10.3390/antiox15080943 (PMC13509557; doi:10.3390/antiox15080943)
Supplement: Supplementary file 1 [file antioxidants-15-00943-s001.zip › Supplementary tables S1-S4.pdf]

Supplementary Table S1. Gensini scoring system used to assess coronary atherosclerotic burden.

**Degree of luminal stenosis**	**Score**	**Coronary segment**	**Weighting factor**
<25%	1	LM	5
25%~49%	2	Proximal LAD	2.5
50%~74%	4	Mid LAD	1.5
75%~89%	8	Distal LAD	1
90%~99%	16	D1	1
100%	32	D2	0.5
		Proximal LCX	2.5
		Distal LCX	1
		OM	1
		Proximal RCA	1
		Mid RCA	1
		Distal RCA	1
		PD	1
		PLV	1

Abbreviations: LM, left main artery; LAD, left anterior descending artery; LCX, left circumflex artery; RCA, right coronary artery; D1, first diagonal branch; D2, second diagonal branch; OM, obtuse marginal branch; PD, posterior descending artery; PLV, posterior left ventricular branch.

Supplementary Table S2. Baseline Characteristics of the Study Participants.

	Normal（n=20）	CAD patients（n=21）		
		**Low**	**Mild**	**High**
Male, n (%)	9 (45%)	7 (87.5) **	5 (100) **	6 (75) *
Age, years	33 ± 5	66.13 ± 15.56**	57.80 ± 9.88**	60.50 ± 9.84**
Heart rate (bpm)	59 ± 10	66.25 ± 13.39	64 ± 4.85	75 ± 14.05
Systolic BP (mmHg)	119.35 ± 13.28	119.13 ± 28.89	96.40 ± 29.31	103.75 ± 36.35
Diastolic BP (mmHg)	70 ± 10	78.13 ± 22.09	85 ± 24.12	99 ± 20.92
Serum creatinine (μmol/L)	56.50 ± 10.27	69.38 ± 13.15	69.20 ± 4.97	80.13 ± 16.81

Data are presented as mean ± SD or n (%). *P < 0.05, **P < 0.01 vs. the Normal group. (by one-way ANOVA with Tukey's post-hoc test for continuous variables; by Chi-square test for categorical variables).

Supplementary Table S3. Comparison of metabolic and inflammatory parameters among study groups.

	Normal（n=20）	CAD Patients（n=21）		
		Low	Mild	High
TC(mmol/L)	3.62 ± 0.91	3.57 ± 0.73	3.86 ± 1.16	4.24 ± 0.94
HDL-C(mmol/L)	1.24 ± 0.30	1.08 ± 0.14	1.04 ± 0.13	0.94 ± 0.21
LDL-C(mmol/L)	2.15 ± 0.43	2.13 ± 0.66	2.25 ± 0.92	2.55 ± 1.59
Atherogenic index(AI)	2.15 ± 1.06	2.52 ± 1.00	2.76 ± 1.14	3.26 ± 1.50
Neutrophils($\times 10^9$/L)	4.11 ± 0.95	4.66 ± 1.35	4.78 ± 1.26	5.44 ± 1.44
Lymphocytes($\times 10^9$/L)	1.77 ± 0.43	0.62 ± 0.21**	1.09 ± 0.59*	1.46 ± 0.66#
Monocytes($\times 10^9$/L)	0.37 ± 0.11	0.34 ± 0.15	0.39 ± 0.14	0.48 ± 0.14
NHR	1.85 ± 0.59	2.04 ± 0.84	2.20 ± 0.86	2.51 ± 1.26**
MHR	0.31± 0.11	0.31 ± 0.13	0.39 ± 0.18	0.53 ± 0.19**
LHR	1.54 ± 0.59	0.73 ± 0.47*	1.09 ± 0.68	1.65 ± 0.57#

Data are presented as mean ± SD. *P < 0.05, **P < 0.01 vs. the Normal group; #P < 0.05 vs. the Low group (by one-way ANOVA followed by Tukey's post-hoc test).

Supplementary Table S4. Exploratory multivariable linear regression analyses for the association between circulating CIRP and Gensini score in patients with CHD.

Model	Adjusted covariates	n	B for CIRP	SE	Standardized β	95% CI for B	p value	Adjusted R^2	VIF for CIRP
Model 1	None	21	0.152	0.045	0.615	0.058-0.246	0.003	0.345	1.000
Model 2	Age	21	0.154	0.046	0.622	0.058-0.250	0.003	0.327	1.003
Model 3	Age, HDL-C, LDL-C	21	0.173	0.063	0.699	0.039-0.307	0.015	0.310	1.889
Model 4	Age, MHR	21	0.204	0.106	0.822	-0.019-0.427	0.071	0.299	5.188
Model 5	Age, SBP, HDL-C, LDL-C, neutrophil count	21	0.213	0.089	0.860	0.023-0.403	0.030	0.238	3.348

Abbreviations: CHD, coronary heart disease; CIRP, cold-inducible RNA-binding protein; HDL-C, high-density lipoprotein cholesterol; LDL-C, low-density lipoprotein cholesterol; MHR, monocyte-to-HDL-C ratio; SBP, systolic blood pressure; SE, standard error; CI, confidence interval; VIF, variance inflation factor.

Table note: Gensini score was used as the dependent variable, and circulating CIRP was used as the main independent variable. B represents the unstandardized regression coefficient. Because of the limited sample size, these multivariable models were considered exploratory and interpreted cautiously.
